# An agent-based model for drug-radiation interactions in the tumour microenvironment: Hypoxia-activated prodrug SN30000 in multicellular tumour spheroids

**DOI:** 10.1371/journal.pcbi.1006469

**Published:** 2018-10-24

**Authors:** Xinjian Mao, Sarah McManaway, Jagdish K. Jaiswal, Priyanka B. Patel, William R. Wilson, Kevin O. Hicks, Gib Bogle

**Affiliations:** 1 Auckland Cancer Society Research Centre, School of Medical Sciences, University of Auckland, Auckland, New Zealand; 2 Maurice Wilkins Centre for Molecular Biodiscovery, University of Auckland, Auckland, New Zealand; 3 Auckland Bioengineering Institute, University of Auckland, Auckland, New Zealand; U. Texas, Austin, UNITED STATES

## Abstract

Multicellular tumour spheroids capture many characteristics of human tumour microenvironments, including hypoxia, and represent an experimentally tractable *in vitro* model for studying interactions between radiotherapy and anticancer drugs. However, interpreting spheroid data is challenging because of limited ability to observe cell fate within spheroids dynamically. To overcome this limitation, we have developed a hybrid continuum/agent-based model (ABM) for HCT116 tumour spheroids, parameterised using experimental models (monolayers and multilayers) in which reaction and diffusion can be measured directly. In the ABM, cell fate is simulated as a function of local oxygen, glucose and drug concentrations, determined by solving diffusion equations and intracellular reactions. The model is lattice-based, with cells occupying discrete locations on a 3D grid embedded within a coarser grid that encompasses the culture medium; separate solvers are employed for each grid. The generated concentration fields account for depletion in the medium and specify concentration-time profiles within the spheroid. Cell growth and survival are determined by intracellular oxygen and glucose concentrations, the latter based on direct measurement of glucose diffusion/reaction (in multilayers) for the first time. The ABM reproduces known features of spheroids including overall growth rate, its oxygen and glucose dependence, peripheral cell proliferation, central hypoxia and necrosis. We extended the ABM to describe in detail the hypoxia-dependent interaction between ionising radiation and a hypoxia-activated prodrug (SN30000), again using experimentally determined parameters; the model accurately simulated clonogenic cell killing in spheroids, while inclusion of reversible cell cycle delay was required to account for the marked spheroid growth delay after combined radiation and SN30000. This ABM of spheroid growth and response exemplifies the utility of integrating computational and experimental tools for investigating radiation/drug interactions, and highlights the critical importance of understanding oxygen, glucose and drug concentration gradients in interpreting activity of therapeutic agents in spheroid models.

## Author summary

Studies in 3D cultures, notably multicellular tumour spheroids that mimic many features of solid tumours, have great potential for speeding up anticancer drug development. However the increased complexity of 3D cultures makes interpretation of experiments more difficult. We have developed a hybrid continuum/agent-based mathematical model, validated by experiments, to aid interpretation of spheroid experiments in developing drugs designed to eliminate radiation-resistant hypoxic cells. This model includes key features of the tumour microenvironment including oxygen and glucose transport and regions of hypoxia where the cells are resistant to radiation, but sensitive to hypoxia-activated prodrugs such as SN30000. This enables us to predict the growth and cell response in untreated spheroids and compare the results to spheroids treated with radiation and SN30000. We demonstrate good prediction of cellular responses in spheroids treated with radiation and SN30000 and good agreement with spheroid regrowth after treatment when additional effects of cellular growth delay are added. This demonstrates that the modelling approach has potential to improve interpretation of experimental investigations of drug and radiation combinations.

## Introduction

Mathematical modelling is gaining increasing attention in the field of cancer research because of advantages such as spatial and dynamic monitoring, visualisation and high-throughput testing [1,2]. In particular, development of 3D multiscale agent-based models that capture key features of the tumour microenvironment have the potential to significantly improve the interpretation of responses to therapeutic agents and to speed up drug development, regimen optimisation and understanding of therapeutic interactions. Several agent-based models that include aspects of the tumour microenvironment have been developed, but few have been applied to drug development, drug/radiation interactions or targeting features of the tumour microenvironment such as hypoxia.

*In vitro* three-dimensional (3D) cell cultures, including multicellular tumour spheroids and multicellular layers (MCLs), capture many features of real tumours, and have advantages over monolayer cell culture for developing drugs. In particular they experimentally model key aspects of the tumour microenvironment that influence therapeutic response including oxygen, nutrient, pH and prodrug/drug diffusion gradients and the resulting microregional variations in gene expression, cell cycle kinetics and cell death [3,4]. MCLs are ideal models for quantifying diffusion and its coupling with reaction in the tumour microenvironment (e.g. prodrug metabolism). Spheroids are desirable for monitoring growth of the whole population of cells over time. Nevertheless, although advances in spheroid culture techniques enable high-throughput production of uniform spheroids, it has proved technically challenging to use spheroids to test complex schedules of drug or radiation combinations and to interpret the data, due to the lack of available non-destructive quantitative endpoints [5,6].

Early mathematical models of avascular tumour or spheroid growth adopted the continuum approximation, giving rise to partial differential equation (PDE) formulations. This approach had the virtues of using well-understood mathematics, and high computational efficiency, but places severe restrictions on the ability to represent important biological features of tumours, in particular their spatial heterogeneity and the diversity of cell fates. An approach that simulates the tumour at the level of individual cells may be better suited to the task of simulating growth, and investigating therapeutic responses [7]. Individual cell fates can be tracked, allowing both short-term cytotoxic and long-term growth delay endpoints to be simulated. Such an approach is variously referred to as agent-based, individual-based, entity-based or cell-based. Agent-based models (ABM) for the growth of tumour spheroids can be roughly classified as on-lattice (this includes the cellular Potts model and approaches often called “cellular automata”) or lattice-free, according to how space is treated. In the on-lattice method, cells are restricted to discrete positions on a regular grid of locations (usually rectangular) [8,9], while the lattice-free method allows continuous cell motion [10–13]. Tumour models are sometimes referred to as “hybrid”, because PDE methods are used to simulate the diffusive transport of constituents (oxygen and nutrients) within the tumour, and “multiscale” because both intracellular processes and the behaviour of the cell population are simulated. In a multiscale model different scales are explicitly hybridised together through feedback methods, thus generating a more complete picture of the biological system than is possible using a method based on one or the other scale alone.

There are several excellent reviews of tumour modelling, discussing the wide range of methods and assumptions adopted: [14–23]. Models of *in vivo* tumours will not be discussed here, except to note that a major factor influencing tumour growth, and complicating the modelling of *in vivo* tumours, is the presence of blood vessels. Growing tumour spheroids *in vitro* enables a level of experimental control of oxygen and nutrients that is impossible with *in vivo* tumours, and correspondingly facilitates the development of a useful model, one with predictive power. Our ultimate goal is a model that can be used to investigate radiation/drug complementarity in anti-cancer therapy. For this reason we limit discussion here to agent-based spheroid models that address killing by radiation or drugs.

Employing a 3D off-lattice model, Kempf et al. investigated spatio-temporal dynamics of spheroid responses to radiation [24,25] using literature values for cell growth rate and oxygen and glucose diffusion parameters. By simulating a cell cycle phase-dependent version of the linear-quadratic (LQ) model for killing by radiation, they showed that cell cycle synchronisation in response to radiation can potentially be exploited through careful timing of dose fractionation. They also investigated reoxygenation after radiation treatment in their model and explored the implications for optimal dose scheduling. Their model predicted rapid partial re-oxygenation followed by recurrence of hypoxia during fractionated radiotherapy regimes. They concluded that full tumour reoxygenation could only be achieved with addition of an efficient hypoxic cell radiosensitiser. They assumed the sensitisation was the same as that for oxygen and did not include drug effects such as diffusion limitations or the drug concentration dependence of sensitisation. In a sequence of papers, Powathil et al. used two ABMs to explore several modes of tumour therapy, with a focus on heterogeneity of hypoxia and cell cycle distribution. With these lattice-based 2D models they investigated how cell-cycle phase heterogeneity leads to differential killing by chemotherapeutic drugs demonstrating that oxygen transport limitations cause heterogeneity in HIF-1 α and cell-cycle status, and that these effects, combined with limited drug transport result in impaired therapeutic efficacy for cell cycle specific drugs[26]. Applying these models they demonstrated that appropriate scheduling can overcome cell cycle phase mediated drug resistance in tumours [27,28]. Notably they demonstrated that appropriate combinations of cell-cycle specific drugs may be used to reduce hypoxia and cell cycle induced resistance during radiation therapy [28,29]. They have also recently extended the model to demonstrate significant radiation-induced bystander effects [30]. Bacevic et al. [31] simulated the effectiveness of adaptive therapy using cyclin-dependent kinase inhibitor drugs exploring the difference in effectiveness observed in monolayers and spheroids. The latter ABM employed to investigate spheroid behaviour is lattice-based and 2D.

When modelling radiation treatment hypoxia is of critical importance as the above studies indicate. [24,28,30] Hypoxia is a common characteristic of many human tumours, attributed to rapid consumption of oxygen and poor oxygen delivery by the disorganised tumour microvasculature [32], and is a well-understood cause of resistance to ionising radiation (radiotherapy) [33,34]. Since hypoxia is more prevalent and severe in tumours than in normal tissues, targeting hypoxia is a promising strategy to improve the therapeutic index of radiation. Hypoxia-activated prodrugs (HAPs) are designed to be minimally toxic until they are activated in regions of very low oxygen concentration. After systemic administration these prodrugs undergo enzymatic reduction by oxidoreductases to active cytotoxic compounds in hypoxic cells in tumours, via intermediates that are re-oxidised by oxygen back to parent prodrugs, hence selectively targeting hypoxic cells [35,36] (For further description of the mechanism of action see Figure J). Therefore, radiation and HAP combination therapy is hypothesised as useful to provide spatial complementarity in the elimination of hypoxic tumour cells that may otherwise reoxygenate and repopulate the tumour after radiation alone.

The activity of HAPs depends on the optimisation of rates of metabolism, to balance loss of the prodrug with generation of the active metabolite [37]. This dependence on metabolism rates makes HAP development a complex undertaking, and calls for experimental and mathematical models that can be used to dissect the pharmacokinetics (PK) and pharmacodynamics (PD) of the prodrugs over spatial scales corresponding to diffusion distances in the tumour microenvironment. SN30000 [38] represents an ideal test compound for development of such tools given that the active cytotoxin is a highly reactive free radical [39] that does not diffuse from the cell in which it is generated [40]. This lack of bystander effects from metabolite diffusion simplifies the ABM, facilitating exploration of radiation and SN30000 combinations in terms of spatial complementarity.

Very few ABM studies have simulated the action of HAPs or their combination with radiation. Kazmi at al. [41,42] developed a 2D on-lattice model based on an artificial neural network representation of cell behaviour [43,44] to investigate the response of hypoxic cells to tirapazamine. This study used literature values of oxygen, glucose and tirapazamine diffusion and metabolism parameters. With a simple categorical model of the cytotoxicity of tirapazamine it was concluded that inner cells would be resistant at exposures well above that achievable *in vivo*, due to limited penetration of tirapazamine.

To explore radiation/HAP combinations, here we develop an on-lattice 3D ABM for the tumour spheroid (spheroid ABM; SABM). Several factors make the agent-based approach essential for this study. While continuum-based methods, typically assuming spherical symmetry, are capable of simulating untreated spheroid growth with a homogeneous cell population, our particular concern is simulation of drug and radiation treatments, both separately and in combination. The probabilistic nature of killing by these treatments inevitably creates an asymmetric spheroid with an irregular pattern of cell death (in contrast to death by hypoxia or starvation.) The presence of voids within the spheroid leads to asymmetry in oxygen and glucose penetration, and in the resulting cell growth rates. Cells destined to die as a result of treatment survive and consume oxygen and glucose until they attempt to divide. In addition we are sometimes interested in simulating a mixed cell population, e.g. with drug-responsive and drug-unresponsive cells. Addressing these issues by any method other than ABM would be extremely challenging. The flexibility of ABMs makes them very suitable for this kind of modelling, and the current study represents a foundation for models that capture additional aspects of tumour biology.

We investigate the ability of the model to predict tumour spheroid growth and responses to radiation and SN30000, both alone and in combination. Cell fate (growth rate, division, death), which is mutually determined by spatially-varying concentrations of oxygen, glucose and therapeutic agents, is tracked as a function of time. Importantly, most of the pivotal parameters of the model were measured using the same human tumour cell line (colorectal adenocarcinoma HCT116), including diffusion and consumption of glucose. The model was first calibrated for growth of HCT116 spheroids, based on measurements of diameter, cell number, viable cell fraction, hypoxic fraction and S-phase fraction of cells, employing flow cytometry and histology. Following that, a pharmacokinetic/pharmacodynamic (PK/PD) sub-model for SN30000 was developed and incorporated into the SABM. The extravascular transport parameters of SN30000 were determined in HCT116 MCL experiments, and the cytotoxicity and metabolism of SN30000 were quantitated in HCT116 stirred cell suspensions. A LQ model of radiation killing was incorporated based on clonogenic cell killing by radiation in HCT116 monolayers under anoxic and oxic conditions, and implemented in the SABM. As an aid to interpretation and parameter fitting of monolayer experiments a monolayer ABM (MABM) was also developed. We report the comparison of simulated and experimental results for clonogenic cell killing and growth delay of spheroids by SN30000 or radiation alone and in combination, under different ambient oxygen levels and point to extensions in progress such as introducing cellular heterogeneity, bystander effects and cell cycle and growth models that are mechanistically linked to oxygen and glucose metabolism.

## Results

### Description of SABM

The model is “hybrid”, in that it combines continuum and agent-based methods. Cells are treated as separate entities, each with its own internal state. Transfers of constituents occur across the cell membrane at a rate determined by the relative intra- and extra-cellular concentrations. Inside the cell rates of change in constituent concentrations of nutrients and drugs are determined by the balance of the trans-membrane transport rates and reaction rates described by ODEs. Oxygen and glucose consumption determines the cell growth rate. A cell divides when its volume reaches a specified threshold, giving rise two cells each with half the volume.

In the extracellular domain, which encompasses the whole volume of medium (as used in the experiment being simulated) together with the extracellular component of the spheroid, concentration fields are determined by solving PDEs for diffusion, with cells acting as sinks for nutrients and sinks for drugs and sources of drug metabolites. Oxygen concentration is specified at the upper boundary of the domain by the gas phase oxygen level.

Cells are constrained to exist at locations on a regular rectangular 3D grid or lattice, which is located in a small cubic region at the bottom of the simulated cell culture well. The only cell motion is that resulting from cell division, when cells are displaced to make space for the new cell.

Cell death can occur as a consequence of oxygen or glucose starvation. Cells are tagged to die after exposure to oxygen or glucose concentrations below a critical concentration for a critical time–these cells die after a time lag. Killing of cells by either drug or radiation is also simulated. The probability of cell death is a function of the radiation dose or intracellular drug concentration. In these cases, tagged cells are non-clonogenic but continue to metabolise nutrients and drugs until they undergo cytolysis when they attempt to divide.

**Fig 1:**
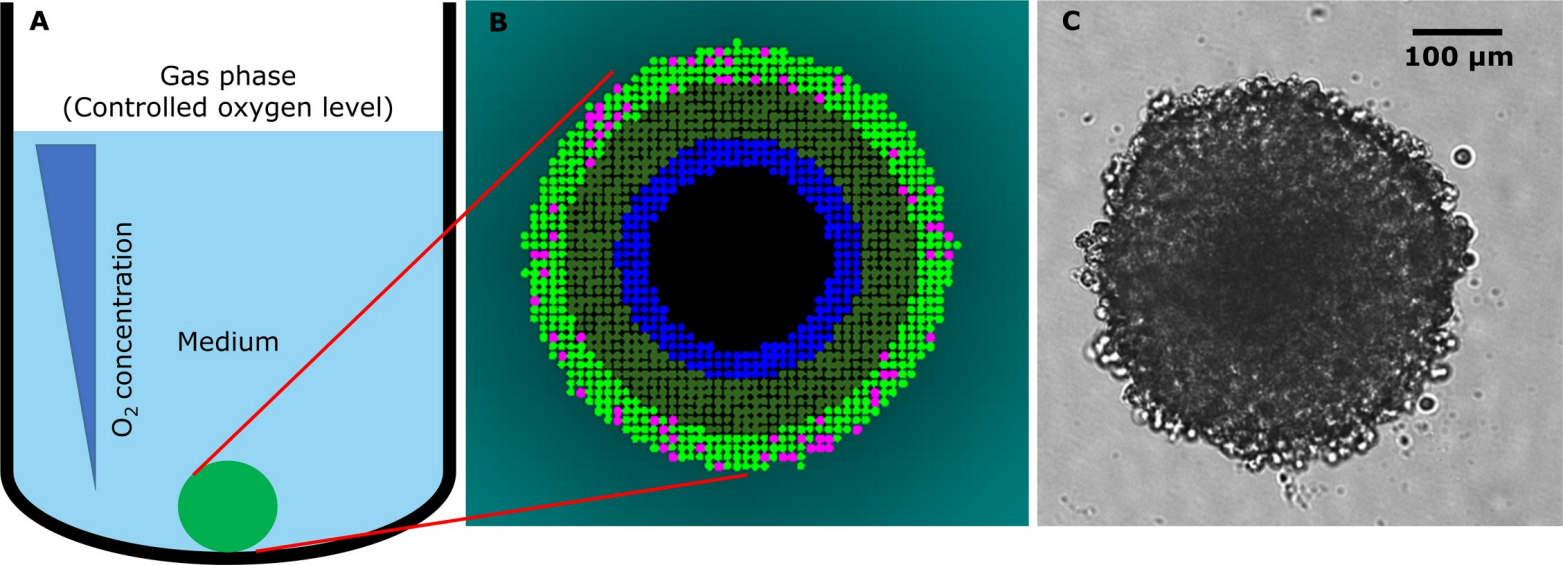
The SABM. (**A)** Cells grow as a blob in a specified volume of medium (0.1–0.2 mL). Cells consume O_2_ and glucose. O_2_ is supplied from the upper boundary and glucose is depleted from the medium unless replenished. Model parameters for diffusion and uptake of constituents, rate of cell growth and death, are acquired from experiments with monolayers and multicellular layers (MCL). Parameters were fitted using the monolayer ABM and Matlab MCL program as described in methods. Time-varying concentration fields of the constituents are solved for in the medium and within the spheroid on a course grid and fine grid respectively (see Figure C), the cells acting as sinks. (**B)** Simulation illustrating cell states as a function of local O_2_ and glucose concentrations. Once cells double in size they undergo mitosis (pink cells). As the spheroid grows, O_2_ and glucose levels decrease in medium near the spheroid (indicated by a cyan color gradient), and decrease in the spheroid core to very low levels with cells becoming hypoxic (< 0.15 μM O_2_, dark green cells). Cells starved of O_2_ or glucose eventually are tagged to die (blue cells) and then, after a delay, undergo cytolysis, creating a necrotic core (black). (**C)** Bright field image of a growing, 4-day HCT116 spheroid seeded at 1000 cells per well.

Simulation of spheroid growth is illustrated schematically in Fig 1 and the model is described fully in the Supplement and Figures A-E.

### Estimation of oxygen and glucose transport parameters

**Fig 2:**
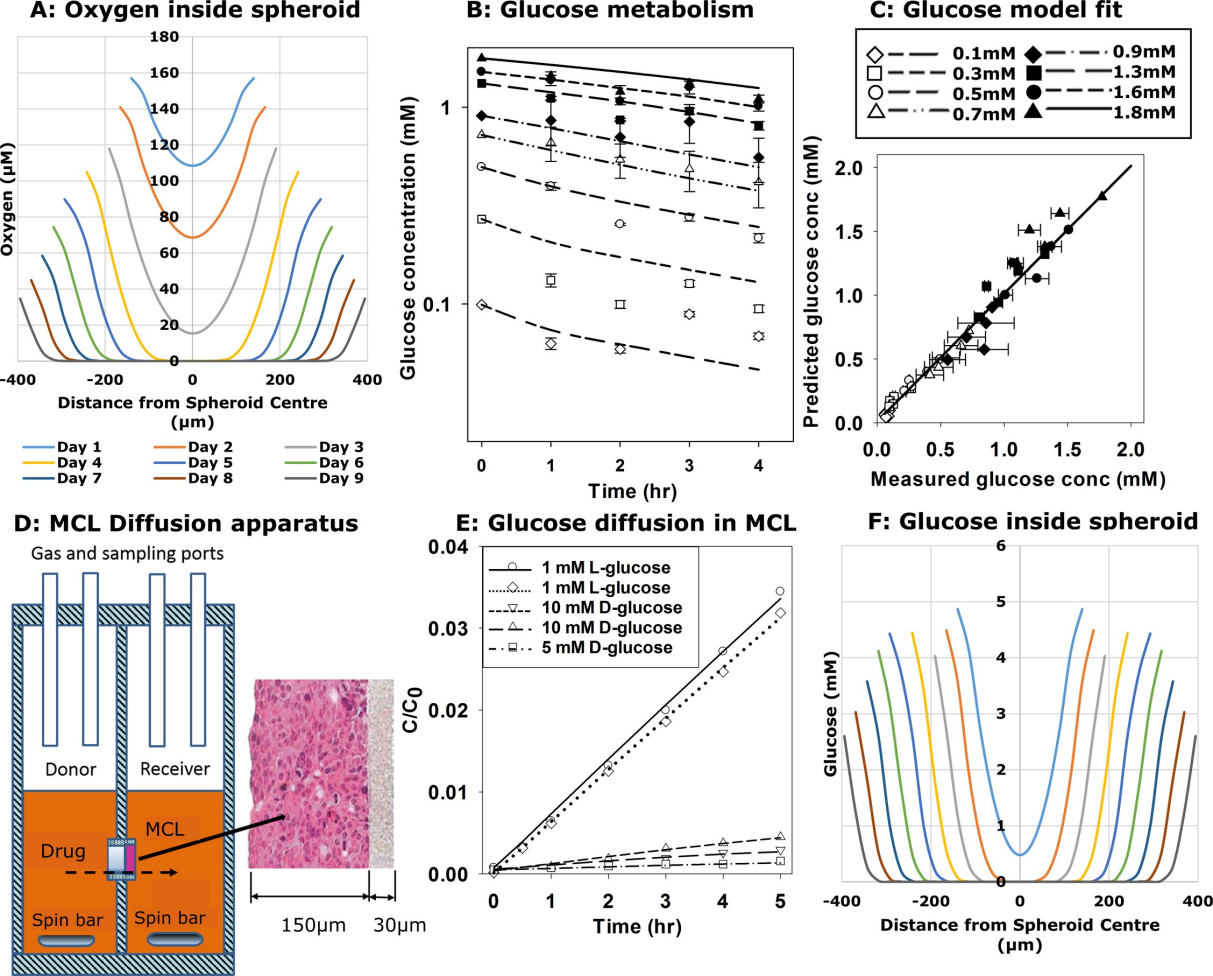
Estimation of oxygen and glucose metabolism and glucose diffusivity. (**A**) Simulated oxygen concentrations as a function of distance from the centre of the spheroid during growth under standard growth conditions of replacing 50% (100 μL) of the culture medium every 2^nd^ day based on experimentally derived oxygen consumption rate and literature medium and tissue diffusion coefficients (see Table 1 and supplementary material). (**B**) Glucose consumption by HCT116 monolayers (10^5^ cells in 0.2 mL/well) in glucose-free medium with no FCS and a range of D-glucose concentrations were measured by serially sampling 5 μL medium at each time point. Values are means ± SD for 4 replicates. D-glucose metabolism rate parameters (*V*_*max*_ and *K*_*m*_) in HCT116 cells were then calculated using the MABM (lines) using parameters that minimize the sum of squared errors between observed and simulated glucose concentrations for the full data set simultaneously. (**C**) Correlation between observed and MABM predicted glucose concentration data in B (R^2^ = 0.977) using the optimized glucose metabolism parameters. (**D**) Schematic figure of apparatus for the measurement of glucose and drug diffusion and metabolism through MCLs grown on a porous support membrane. After drug is added to the donor compartment, samples were collected from the donor and receiver compartments at intervals for measurement of concentrations of glucose or drug and its metabolite(s). (**E**) Representative diffusion data showing transport of ^3^H-L-glucose or D-glucose through HCT116 MCL (grown for 3 days, ca 100 μm thickness). Concentrations are normalised to the initial concentration measured in the donor compartment. Lines are model fits (minimization of the sum of squared errors between observed and calculated concentrations) with fitted parameters *V*_*max*_ for D-glucose, after fixing the glucose coefficient at its mean value determined for L-glucose curves. (**F**) Simulated D-glucose concentration as a function of distance from the centre of the spheroid during growth under standard growth conditions of replacing 50% (100 μL) of the culture medium every 2^nd^ day.

To simulate spheroid growth we first estimated the transport parameters of O_2_ and glucose for HCT116 cells (Fig 2). Oxygen and glucose concentration-distance profiles during spheroid growth from the SABM based on the final parameters in Table 1 are also shown in Fig 2. Oxygen penetration has been well studied, and is known to be limited by its metabolic consumption [45]. We determined the oxygen consumption rate in log-phase HCT116 monolayers, using a Seahorse flux analyser, as (6.35 ± 0.42) × 10^−17^ mol cell^-1^ s^-1^, which was similar to that previously measured in our lab [46] and reported in the literature [47]. Based on accepted estimates of the diffusion coefficient of 2 x 10^−5^ cm^2^ s^-1^ [48] and *K*_*m*_ of 1.33 μM [49] in respiring tissue (this is the Michaelis-Menten parameter for cellular oxygen consumption rate as a function of oxygen concentration), the ABM simulates a penetration distance of ca 125 μm to reach the critical level of 0.15 μM O_2_ for long term survival of cells (see below) in HCT116 day 4 spheroids of 460 μm diameter under standard growth conditions (20% O_2_ in the gas phase, Fig 2A).

**Table 1:** Key parameters in the ABM. The parameters used in the ABM are summarized here, including parameter name (units), value and source (either from literature, assumption, or measurement from experiment).

Parameter	Unit	Value	Source	Meaning & comments
**Cell division**				
T_d monolayers_	hr	19	Measured^*a*^, Figure F	Median monolayer cell division time
T_d spheroids_	hr	22	Measured^*a*^, Fig 5A	Median spheroid cell division time
φ		0.5	Ref [50]	Cell volume fraction
V_HCT116_	pL	1.2	Measured	Median volume of HCT116 cell
Divide size	pL	1.6	Assumed	Volume of dividing HCT116 cell
Divide variation	pL	±0.3	Assumed	Uniformly distributed variation of volume of dividing HCT116 cell
**Oxygen**				
D_O2_	cm^2^ s^-1^	2×10^−5^	Ref [48]	Spheroid oxygen diffusion coefficient
D_medium-O2_	cm^2^ s^-1^	5×10^−5^	Assumed	Medium oxygen diffusion coefficient
V_max-O2_	mol cell^-1^ s^-1^	(6.35 ± 0.423) ×10^−17^	Measured^*a*^	Max oxygen consumption rate (OCR)
K_m-O2_	μM	1.33	Assumed	Michaelis-Menten K_m_ of oxygen
Tag-conc_oxygen_	μM	0.15	Assumed	Tag threshold oxygen concentration for hypoxia
Tag-time_oxygen_	hr	24	Measured^*a*^, Figure G	Tag time limit of hypoxia–cell tagged after this time below threshold
Death-delay_oxygen_	hr	24	Measured^*a*^, Figure G	Death delay time for hypoxia–cell dies following this delay after tagging
**Glucose**				
D_glucose_	cm^2^ s^-1^	(2.08 ± 0.12) ×10^−7^	Measured^*a*^, Fig 2E	Spheroid glucose diffusion coefficient estimated from L-glucose diffusion in MCL
D_medium-glucose_	cm^2^ s^-1^	(8.74 ± 0.19) ×10^−6^	Measured^*a*^	Medium diffusion coefficient of glucose estimated from D-glucose diffusion in microporous support membranes
V_max-glucose_	mol cell^-1^ s^-1^	(7.12 ± 0.82) ×10^−17^	Measured^*a*^, Fig 2B	Max consumption rate of glucose estimated from D-glucose transport in MCL
K_m-glucose_	μM	92	Measured^*a*^, Fig 2B	Michaelis-Menten K_m_ for glucose estimated from monolayer glucose metabolism experiments.
Tag-conc_glucose_	μM	0.15	Assumed	Tag threshold glucose concentration for starvation
Tag-time_glucose_	hrs	48	Measured^*a*^	Tag time limit of glucose starvation
Death-delay_glucose_	hrs	24	Measured^*a*^	Death delay time for glucose starvation
**SN30000**				
D_SN_	cm^2^ s^-1^	(1.17 ± 0.52) ×10^−6^	Measured^*a*^, Fig 6C	Spheroid diffusion coefficient estimated from SN30000 transport experiments under 95% O_2_ to suppress bioreductive metabolism
D_medium—SN_	cm^2^ s^-1^	(7.52 ± 0.13) ×10^−6^	Measured^*a*^	Medium diffusion coefficient of SN30000 estimated from diffusion in collagen coated support membrane membranes
*K*_*in*_	min^-1^	10	Assumed	Cell influx rate of SN30000
*K*_*out*_	min^-1^	10	Assumed	Cell efflux rate of SN30000
K_met0 (cells)_	min^-1^	1.88	Measured^*a*^, Fig 6A	Intracellular rate constant for metabolism in monolayer
K_d_	mM^-2^	85	Measured^*a*^, Fig 6B	kill probability constant of SN30000
K_met0 (MCL)_	min^-1^	1.61 ± 0.18	Measured^*a*^, Fig 6C	intracellular rate constant for metabolism in MCL
K_O2_	μM	1.14	Ref [38]	the oxygen concentration at which SN30000 metabolism is half-maximal
F_2_		1	Ref [38]	The fraction of SN30000 cellular metabolism that is oxygen dependent
**Radiation**				
*α*_*H*_	Gy^-1^	0.189 ± 0.003	Measured^*a*^, Fig 7A	α radiosensitivity parameter under hypoxia in LQ model
*β*_*H*_	Gy^-2^	0.0061 ± 0.0007	Measured^*a*^, Fig 7A	β radiosensitivity parameter under hypoxia in LQ model
*OER*_*α*_, *OER*_*β*_		2.64 ± 0.23	Measured^*a*^, Fig 7A	Oxygen enhancement ratio (α) in LQ model (*OER*_*α*_and *OER*_*β*_were fixed at the same value in parameter fitting)
K_ms_	μM	4.28	Ref [51]	K_m_ for radiosensitivity, the oxygen concentration at which radiosensitivity is half-maximal
*P*_*D*_, Death probability		1 (no delay), 0.6 (with delay)	Assumed	Cell death probability of cells tagged by radiation in each cell cycle
*GD*, growth delay factor	hr Gy^-1^	0 (no delay), 3 (with delay)	Assumed	Cell growth delay induced by radiation
*N*_*GD*_, GD cycles		0 (no delay), 4 (with delay)	Assumed	Number of cell cycles for which cell growth rate is delayed by radiation

^*a*^ Measured in the present study

In contrast, penetration of D-glucose (which we take to be the sole nutrient in the SABM) through metabolically active tissue is less well understood. Glucose consumption by HCT116 monolayers under 20% O_2_ (Fig 2B and 2C) was well fitted using a single Michaelis-Menten term in the monolayer model (MABM), providing estimates of *V*_*max-glucose*_ = 9.0 × 10^−17^ mol cell^-1^ s^-1^ and *K*_*m*_ = 92 μM. The fitting was carried out on the results of 8 monolayer cell culture experiments in which the initial medium glucose concentration was: 0.10, 0.27, 0.50, 0.72, 0.90, 1.32, 1.51, 1.77 mM. In each case oxygen (at atmospheric level) was not limiting, and the initial cell number was 95000/well. Medium glucose levels were measured at 1, 2 and 4 h, giving a total of 24 observations. The objective function was the sum of squares of errors, where error = (observation–simulated value). Experiments were conducted to measure the diffusion coefficient of glucose in HCT116 MCL (Fig 2D). Transport of ^3^H-L-glucose, which is neither transported into nor metabolised by cells, was used to estimate the extracellular diffusion coefficient of glucose (*D*_*glucose*_) through MCLs (Fig 2E), assuming that *D*_*glucose*_ of both optical isomers of glucose (L-glucose and D-glucose) are the same. L-glucose transport was more rapid than that of D-glucose through MCLs of similar thickness, consistent with rapid cellular consumption of the latter (Fig 2E). In these studies, the thickness of each MCL was determined by diffusion of co-administered ^14^C-urea, and the glucose diffusion coefficient in the microporous Teflon support membrane (*D*_*sup-glucose*_) was fixed at the mean value determined for D-glucose in separate experiments using bare support membranes without a MCL present. Fitting the L-glucose concentration-time data using our Matlab program [50] to solve the glucose diffusion equations (supplementary material) without reaction gave *D*_*glucose*_ = (2.08 ± 0.12) × 10^−7^ cm^2^ s^-1^. This allowed *V*_*max-glucose*_ to be estimated from the D-glucose transport data (Fig 2E), assuming the same K_m_ as for single cell (92 μM, Fig 2B), and a cell volume fraction of 0.5 in MCLs as previously determined [50]. The resulting value of *V*_*max-glucose*_ = (7.12 ± 0.82) × 10^−17^ mol cell^-1^ s^-1^ was similar to that estimated in monolayers and was used in subsequent SABM simulations. The estimated penetration distance for D-glucose to decrease to 0.15 μM was 120 μm, similar to that of oxygen (Fig 2F). Due to the surprisingly low glucose diffusion coefficient, glucose concentration profiles were steep, but with the current parameters glucose had little effect on simulated spheroid growth or cell death under these well-fed conditions (see Discussion).

The glucose consumption model was then tested by growing monolayers without medium changes and measuring cell number (Figure Fa) and glucose concentrations (Figure Fb) over 10 days. In these experiments the estimated doubling time (19 hr) in glucose-replete medium was well-fitted by the MABM without further parameter adjustment (Figure Fa), with growth rates falling when glucose concentrations fell below 2 mM.

### Comparison of SABM with histological characteristics of spheroids

**Fig 3:**
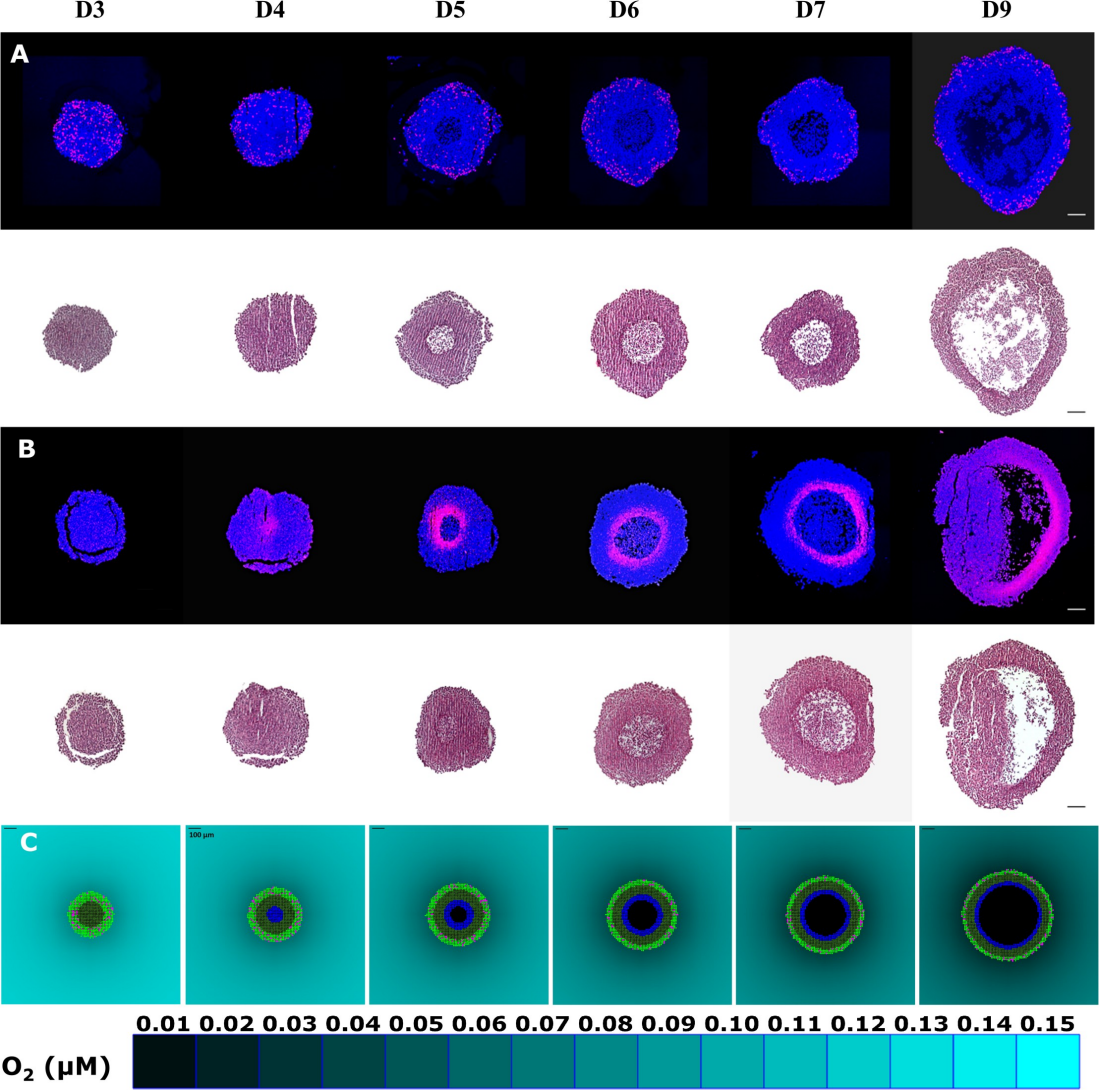
Histological characteristics of spheroids visualised in central sections and comparison with outputs of the SABM. S-phase cells in spheroids collected after the indicated days of growth were stained after incubation with EdU (panel **A**). Hypoxic cells in spheroids were visualised by immunostaining for EF5 binding (panel **B**). The same spheroid sections were stained with H&E to quantify the necrotic and viable rim size (lower panels of **A**, **B**). n = 48 spheroids for **A** and **B**, respectively. Panel **C** shows SABM simulations based on oxygen and glucose parameters fitted to monolayer ad MCL experimental data (See Table 1). 2D snapshots of a central plane through spheroids are shown in which pink cells represent dividing cells, light green cells are highly proliferative cells under well oxygenated microenvironment (>1 μM O_2_), dark green cells are slowly proliferative cells under hypoxia (<1 μM O_2_), blue cells have been hypoxic for > 24 hr and have been tagged for subsequent necrosis, and the black region in the centre represents the necrotic zone. Scale bars = 100 μm and a color-coded legend for oxygen concentration in the medium is shown.

**Fig 4:**
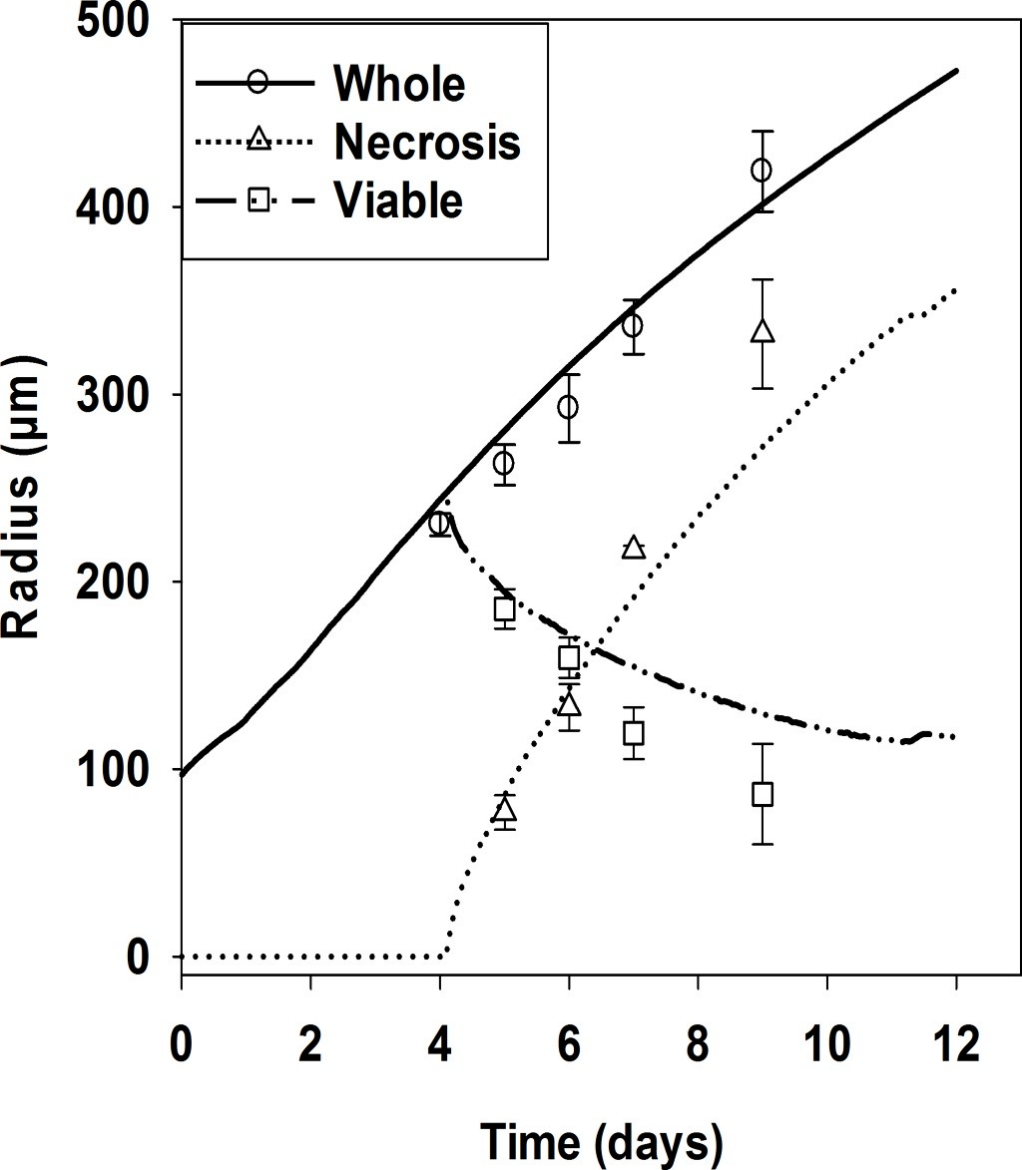
Quantitation of histological characteristics of spheroids and comparison with outputs of the SABM. The overall diameter (circles), diameter of the necrotic region (triangle), and thickness of viable rim (plotted here as twice the viable rim thickness, rectangles) of HCT116 spheroids were quantitated using H&E stained histological sections illustrated in Fig 3. Values are means ± SD (n = 4 spheroids, 5 on day 7). The lines are the corresponding outputs of SABM simulations using oxygen and glucose parameters fitted to monolayer and MCL experimental data (See Table 1).

As described in the Supplement, cells in the SABM grow at a rate proportional to the oxygen consumption and glucose consumption rates, with maximum growth rate determined by input of the maximum doubling time of cells in spheroids (T_d spheroids_ adjusted to 22 hr). Predictions of the SABM, for well-fed spheroids (partial medium replacement every two days) grown under 20% O_2_, were compared to histological spheroid sections. S-phase cells (identified by incorporation of the thymidine analogue 5–ethynyl–2′–deoxyuridine, EdU), hypoxic cells (identified by covalent binding of the hypoxia probe EF5), necrotic and viable rim diameters are illustrated by representative images of spheroid sections in Fig 3. S-phase cells were evenly distributed within spheroids on day 3 (top panel of Fig 3A), with no central hypoxia (top panel of Fig 3B). By day 4, a gradient of EdU positive cells was observed from the periphery to the centre of spheroids, consistent with limitation of nutrient and oxygen supply. Hypoxia but not necrosis (bottom panel of Fig 3A and 3B) was found on day 4, indicating that the central cells at this stage were hypoxic but still viable. Necrosis was observed in the core of spheroids on day 5 and increased as a function of time (bottom panel of Fig 3A and 3B), while the thickness of the viable rim of spheroids increased before day 4, followed by a gradual decrease from day 5. To represent the sharp delineation between the viable hypoxic cells and necrosis, time dependent cell death due to severe hypoxia (evaluated experimentally in Figure G) was represented in the SABM by tagging cells as fated to die if they experience < 0.15 μM O_2_ for 24 hr with a further delay of 24 hr before cellular necrosis. Using the finalised parameters of the SABM (Table 1), the model recapitulated the histological features with hypoxia occurring as a rim between the central necrotic region and proliferative cells in the periphery (Fig 3C). Quantitatively, the model simulated the relationship between the thicknesses of spheroid, necrosis and viable rim well (Fig 4).

### Comparison of SABM with flow cytometry profiles of spheroid cells

**Fig 5:**
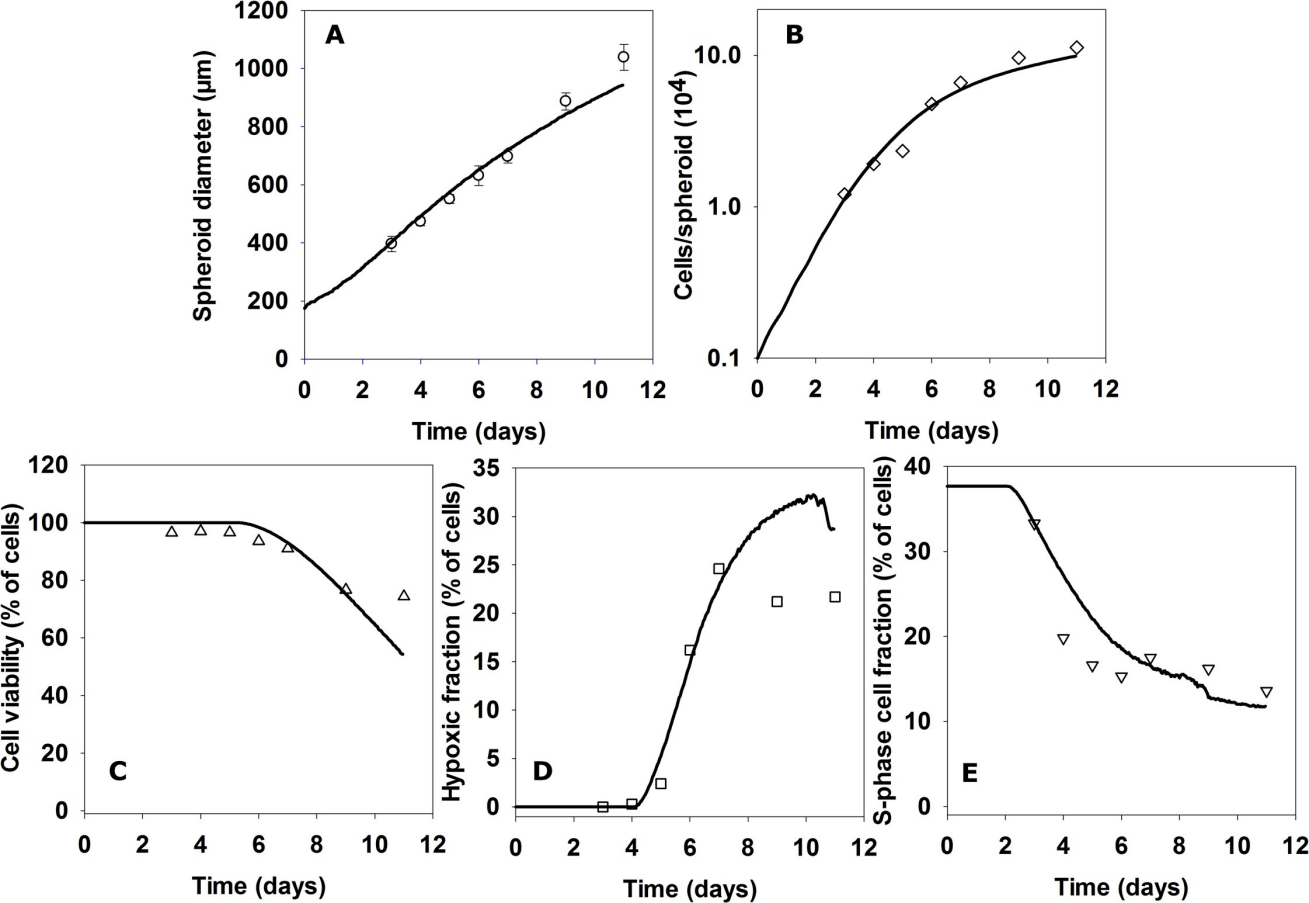
Comparison of growth and cellular characteristics of HCT116 spheroids with outputs of the SABM. On the indicated days, diameters of spheroids (**A**) were measured. Values are means ± SD (n ≥48 spheroids). After exposing spheroids to EdU or EF5, spheroids (n = 48 spheroids) were pooled and dissociated and the number of cells in cell suspension were counted to estimate total cell number per spheroid (**B**), followed by exposing cells in 1 μg/mL PI for 2 min to measure cell viability by flow cytometry (% PI negative, **C**). Hypoxic fraction (% EF5-positive cells, **D**) and S-phase fraction (% EdU-positive cells, **E**) were measured by flow cytometry. The predictions of the SABM (lines), using fitted parameters from monolayer and MCL experiments, plotted with experimental data (points) are: (A) spheroid diameter (A) total cell number, (B), fractions of cells not tagged for death (C), fraction of cells below 0.5μM O_2_ (D) and estimated S-phase fraction based on the assumption that 37% of cells were initially in S-phase.

In a separate experiment total cell numbers and subpopulations of viable (propidium iodide (PI)-negative), S-phase (EdU-positive) and hypoxic (EF5-positive) cells were quantified by flow cytometry (FCM) after enzymatic dissociation of HCT116 spheroids (Fig 5). Overall diameters of spheroids were again well-predicted by the SABM (Fig 5A). The SABM also predicted total cell number in spheroids as exponentially increasing until day 4, then a gradually decreasing relative rate reaching about 9 × 10^5^ cells per spheroid on day 11 (Fig 5B). Representative FCM distributions are illustrated in Figure H. Cell viability (simulated by the number of cells not tagged for death by hypoxia) was well predicted over this period (Fig 5C) when compared to cell viability by PI staining. The hypoxic fraction increased after day 4 (Fig 5D) while the proportion of S-phase cells decreased (Fig 5E), as observed in the above histology study. These time trends were again well predicted by the SABM, assuming that the initial S-phase fraction in small spheroids was 38%. Overall, the simulated growth kinetics and cellular characteristics of spheroids agreed well with experiments when tag-concentrations for oxygen and glucose were both set at 0.15 μM with the lag between tagging and necrosis as reported in Table 1.

### Oxygen and glucose dependence of spheroid growth

In the SABM, cell growth rate is proportional to cell metabolic rate, which is proportional to the product of the rates of metabolism of glucose and oxygen (Eqn S10, Supplement). Good agreement was found between simulated and experimental results in terms of oxygen dependence of spheroid growth, with progressive decreases in growth rate when ambient O_2_ was lowered from 20% to 5% and 1% (Figure Ia). With the D-glucose metabolism and diffusion parameters determined above, the model gave good predictions for spheroid growth in medium without D-glucose supplementation (Figure Ib), and for D-glucose consumption in medium (Figure Ic). Spheroids cultured in medium without supplementation (Figure Ib) showed only slightly slower growth rates than well-fed spheroids (Fig 5A) until day 10 when D-glucose in the medium was almost exhausted (Figure Ic).

### Development of PK/PD model of SN30000

**Fig 6:**
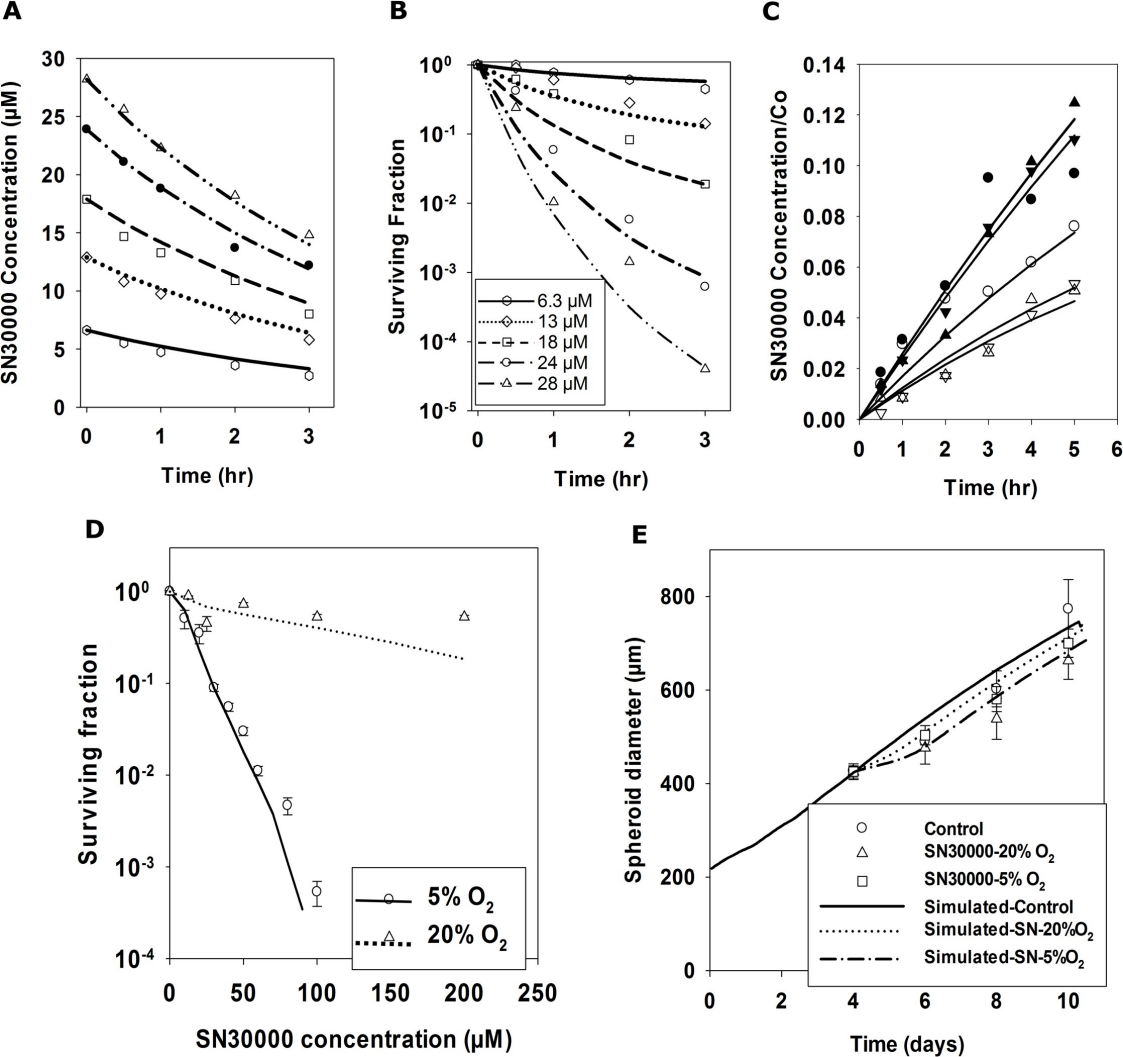
Development of a spatially resolved PK/PD model for SN30000 and testing by SABM simulation of spheroid response to SN30000 exposure. Cellular bioreductive metabolism of SN30000 (**A**) and clonogenic cell killing (**B**) was measured by serial sampling of stirred HCT116 cell suspensions (2 × 10^6^ cells/mL) under anoxia (0% oxygen gas mixture). The rate constants for SN30000 metabolism (*k*_*met0*_) and cell killing (*K*_*c*_) were estimated by fitting the data in panel **A** and panel **B** simultaneously using the MABM assuming the medium was fully stirred. Lines represent MABM predictions based on the parameters which minimize the overall error sum of squares (Table 1). (**C**) Diffusion parameters for SN30000 were estimated from HCT116 MCL transport studies illustrated for a single experiment. Lines are model fits using the reaction-diffusion program described in methods with fitted parameters for each MCL: D_SN30000_ estimated from MCL transport under hyperoxia (95% O_2_, filled symbols in panel **C**) assuming no metabolic consumption (confirmed by no production of SN30000-1-oxide); *k*_*met0*_ estimated from SN30000 diffusion under anoxia (open symbols in panel **C**) by fixing *D*_SN30000_ at its mean value (Table 1) determined in hyperoxic experiments. (**D**) To quantitate cytotoxicity, spheroids seeded with 1000 cells were exposed on day 4 to a range of SN30000 concentrations for 2 hr under 20% or 5% O_2_ and clonogenic cell survival was measured (points in Panel **D**) and compared to predictions of SABM incorporating the optimized reaction-diffusion and cell kill parameters estimated from monolayer and MCL experiments from Table 1 (lines in Panel **D**). (**E**) To measure spheroid growth delay induced by SN30000, spheroids were exposed to 25 μM SN30000 under 5% O_2_ or to 100 μM SN30000 under 20% O_2_ for 2 hr and spheroid diameters were monitored (points in Panel **E**) and compared to the SABM predictions (lines in Panel **E**) using parameters from Table 1. Values are mean ± SD (n = 16 spheroids).

Experiments were performed with HCT116 stirred cell suspensions to assess the parameters in the PK/PD model that relate clonogenic cell killing to exposure to SN30000 and its metabolic activation, as illustrated for one of three experiments in Fig 6A and 6B. As previously for other cell lines [38], SN30000 was rapidly metabolised in HCT116 cell suspensions under anoxia (Fig 6A), with formation of its 1-oxide metabolite (Figure Ka) confirming that loss of SN30000 is due to bioreductive metabolism. The MABM was used to simultaneously estimate the intracellular rate constant for bioreductive metabolism under anoxia (*K*_*met0*_ = 1.88 min^-1^ Eqn S14) by fitting SN30000 concentrations (Fig 6A) and the kill probability constant (*K*_*d*_ = 85) using kill model 2 (Eqn S19) by fitting to the time dependent clonogenic data (Fig 6B). This simultaneous fitting compensates for loss of SN30000 from the medium by cell metabolism during the experiments. Cell kill model 2 relates loss of clonogenicity to SN30000 concentration and metabolism as previously observed [38], consistent with its proposed mechanism of action (Figure J). Membrane transport parameters were fixed at arbitrary high values, assuming this is not rate limiting and the fitting criterion was minimisation of the overall sum of squared errors.

SN30000 transport through HCT116 MCLs under hyperoxic (95% O_2_) conditions (Fig 6C) was rapid (about 2-fold that of co-administered ^14^C-urea) with an estimated diffusion coefficient of SN30000 (D_SN_) = (1.17 ± 0.52) × 10^−6^ cm^2^ s^-1^, similar to that previously estimated for HT29 MCLs [38]. Transport of SN30000 under anoxic conditions was reduced, consistent with its bioreductive metabolism (Fig 6A), from which the rate constant of SN30000 metabolism (*K*_*met0(MCL)*_) was estimated as 1.79 ± 0.18 min^-1^ fixing the intracellular volume fraction in HCT116 MCLs at 0.5 as previously [50]. The latter is in good agreement with the rate constant determined above using the MABM for anoxic single cell suspensions (Fig 6A).

The cellular PK/PD model for SN30000 (Eqns S14 and S19) was incorporated into the SABM. When a SN30000 dose is simulated, cells are randomly tagged for SN30000-induced clonogenic cell death as a function of concentration, time and O_2_ concentration, and cells undergo cytolysis at the next attempted cell division if they reoxygenate sufficiently for cell growth to proceed. The oxygen dependence parameters for SN30000 cell metabolism were assumed from previous measurements that determined the oxygen concentration at which metabolism is half-maximal in HT29 cells (K_O2_ = 1.14 μM) and demonstrated that there is no significant oxygen-independent metabolism (F_2_ = 1 in Eqn S14) [38]. However, this previous model for SN30000 over-estimated cell killing in stirred suspensions of HCT116 cells under 20% O_2_; consequently the Hill coefficient, N, for oxygen dependence of cell killing was estimated as 1.7 from cell kill data for HCT116 cells exposed to SN30000 under 20% O_2_ (Figure L).

Given that the cytotoxicity of SN30000 and radiation is highly dependent on O_2_ concentration, we note that the hypoxic fraction of spheroids under 5% O_2_ (Figure Ma) predicted by the SABM was significantly higher than that under 20% O_2_ (Fig 3C). In agreement, the hypoxic fraction of 4 day spheroids, assessed by EF5 flow cytometry, was substantially higher after transfer to of spheroids to 5% O_2_ (Figure Mb) than that under 20% O_2_ (Fig 5D). This was also confirmed by histological images of spheroid sections stained by EF5 (Figure Mc versus Fig 3B). Using the above SN30000 transport and cytotoxicity parameters, PD responses of spheroids exposed to SN30000 under 5% or 20% O_2_ were simulated and compared to clonogenic cell survival (Fig 6D) and growth delay (Fig 6E) of HCT116 spheroids. As expected, SN30000 showed limited cytotoxicity in spheroids exposed under 20% O_2_, while its cytotoxicity under 5% O_2_ was significantly higher, demonstrating hypoxia selectivity (Fig 6D). The corresponding simulations predicted the experimental data (lines in Fig 6D). Figure N demonstrates the predicted O_2_, glucose and SN30000 concentration profiles under 5% O_2_ together with SN30000 profiles under 95%, 20% and 0% when spheroids are exposed to 25 μM SN30000, showing a marked oxygen-dependent decrease in SN30000 concentration at the spheroid centre. The model also successfully predicted that there is only a small spheroid growth delay due to SN30000 under 20% or 5% O_2_ (Fig 6E) as a result of limited killing of the well-oxygenated proliferating cells in the spheroid rim.

### Parameterisation of a SABM LQ radiation model

**Fig 7:**
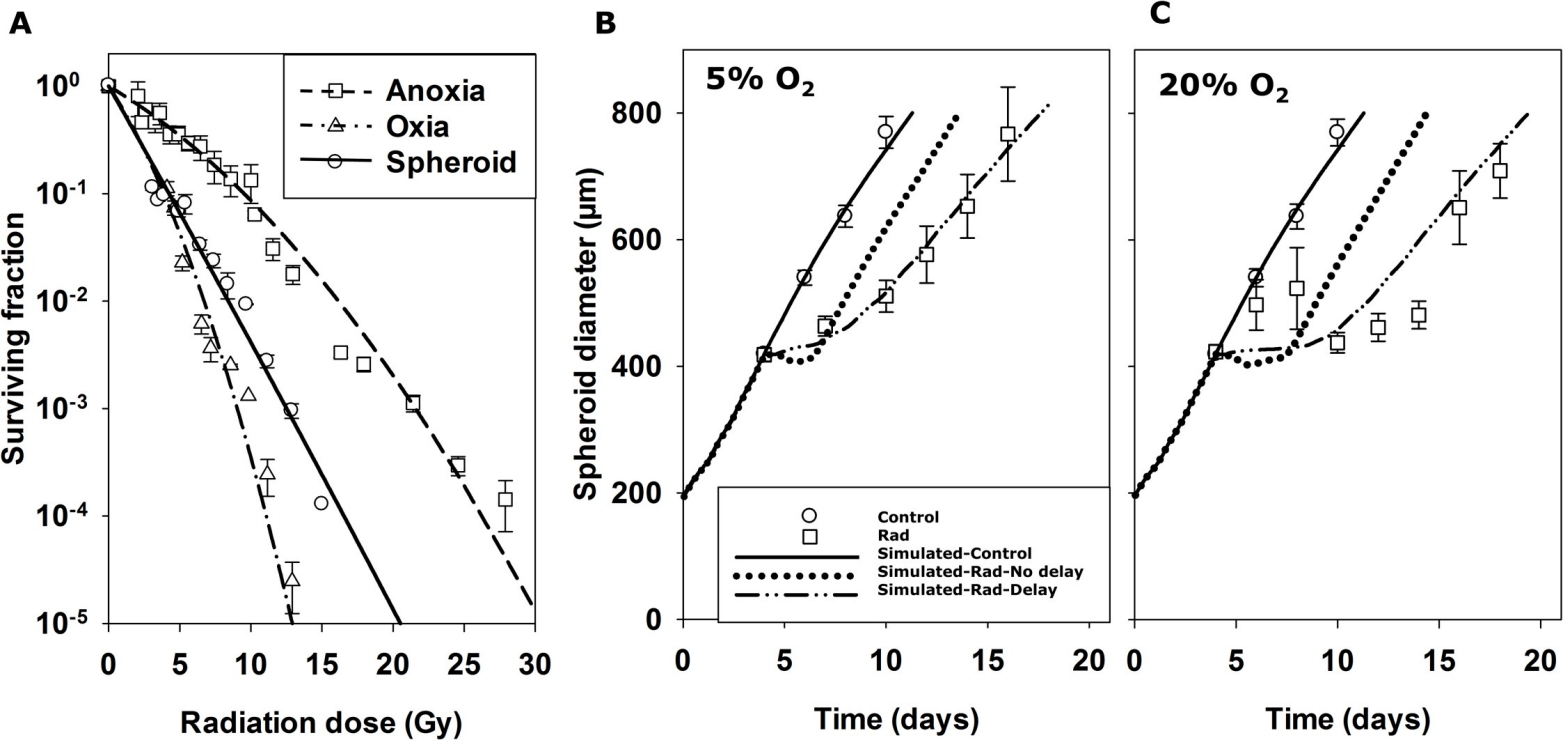
Radiation model and simulation of spheroid response to radiation using the SABM. (**A**) A LQ radiation model was parameterised by measuring clonogenic cell survival of HCT116 monolayer (10^5^ cells/mL) in response to a range of radiation doses under anoxic (unfilled rectangles in **A**, 3 separate experiments) or oxic (unfilled triangles in **A**, 2 separate experiments) conditions. Values are means ± SEM. Lines represent LQ model fits (Eqn S12) to all monolayer data simultaneously (fitted parameters in Table 1) by minimization of the sum of squared errors between simulated and observed ln(SF). These parameters were used in the SABM to simulate clonogenic cell survival of cells in 4-day spheroids exposed to radiation under 20% O_2_ (solid line in **A**) and compared to measured clonogenic cell killing in HCT116 spheroids (filled circles in **A**). Values are means ± SD (n = 16 spheroids). To compare the measured spheroid growth delay induced by radiation to that predicted by the SABM, HCT116 spheroids were exposed to 4 Gy radiation under 5% (Panel **B**) or 20% O_2_ (Panel **C**) and spheroid growth was monitored as a function of time. Values are means ± SD (n = 16 spheroids) and lines are simulations by the SABM based on clonogenic cell killing alone (^…^), or with addition of parameters for cell growth inhibition and survival probability at each mitosis following radiation (—) as described in Table 1.

We further tested the predictive ability of the SABM by evaluating pharmacodynamic responses (cell killing and growth delay) of radiation-treated spheroids. To parameterise a model for radiation sensitivity, HCT116 monolayers were exposed to a range of radiation doses under oxic and anoxic conditions and clonogenic cell survival was measured (Fig 7A). A LQ equation (Eqn S12) was fitted to estimate coefficients α_H_ (0.186 ± 0.003 Gy^-1^) and β_H_ (0.0061 ± 0.0007 Gy^-2^) under anoxia, together with a single maximum oxygen enhancement ratio (OER_αmax_ = OER_βmax_ = 2.63 ± 0.23, Eqn S13) to account for increased radiosensitivity of HCT116 monolayers under oxic conditions (Fig 7A). The SABM incorporated this LQ model at a range of oxygen concentrations using the previously published *K*_*m*_ for radiosensitivity (*K*_*ms*_, oxygen concentration at which radiosensitivity is half-maximal) of 4.28 μM O_2_ [51]. When radiation is simulated, cells are randomly tagged for radiation-induced clonogenic cell death as a function of dose and O_2_ concentration, and undergo cytolysis at the next attempted cell division (post-mitotic cell death). The model successfully predicted the clonogenic cell killing by radiation of cells in spheroids under 20% O_2_ (Fig 7A), based on the parameters derived from HCT116 monolayers described above and spheroid growth and nutrient parameters in Table 1.

**Fig 8:**
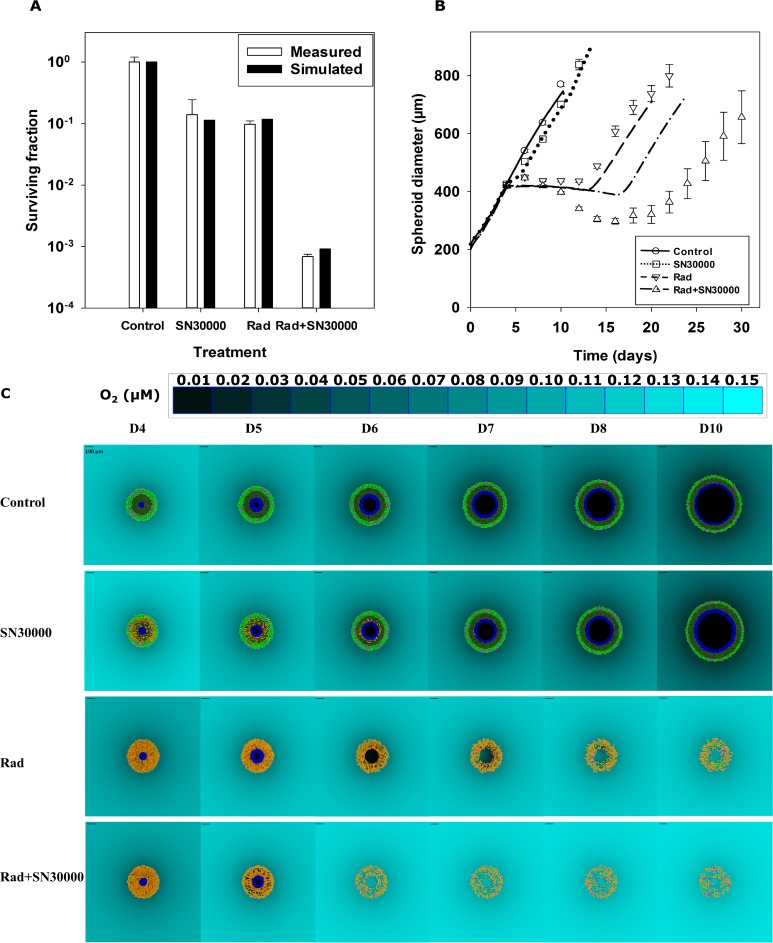
Comparison of simulated and measured spheroid responses to SN30000 and radiation in combination. The predicted and measured clonogenic cell killing (**A**) and growth (**B**) of spheroids seeded with 1000 cells and treated on day 4 under 5% ambient O_2_ with 25 μM SN30000 for 2 hr alone, 12 Gy radiation alone or both (25 μM SN30000 for 2 hr, followed by 12 Gy radiation 1 hr after SN30000 removal). Values are means ± SEM (n = 8 spheroids for **A** and n = 16 spheroids for **B**). SABM predictions are based on the optimized parameters in Table 1. 2D snapshots from the SABM (**C**) illustrate cell fate and spheroid regrowth after the treatments, with the D4 output immediately following irradiation. Yellow cells are tagged for treatment-induced death, pink cells represent dividing (mitotic) cells, light green cells were highly proliferative well-oxygenated cells, dark green cells were low proliferative hypoxic cells, blue cells are cells tagged for hypoxia-induced cell death, and central dark region represents necrosis. Cell motility and fluid flow, to simulated spheroid shrinkage, is not implement yet (see text). The scale bar = 100 μm and a color-coded legend for oxygen concentration in medium is shown.

To investigate spheroid growth delay induced by radiation, spheroids were treated with or without 4 Gy radiation under 5% or 20% O_2_ on day 4 and spheroid diameters were measured as a function of time. Radiation-induced spheroid growth delay was lower for spheroids exposed to radiation under 5% O_2_ (Fig 7B) than under 20% O_2_ (Fig 7C). Based on clonogenic cell killing alone, the SABM poorly predicted radiation-induced spheroid growth delay, predicting rapid regrowth after rapid cytolysis and reoxygenation. This occurred despite good prediction of clonogenic cell killing by radiation in spheroids (see also Fig 8), To address this issue, delays in cell proliferation and cytolysis induced by radiation, similar to those in [17], were introduced: death probability (probability of cytolysis at each cell division), growth delay factor (delay in cell growth modelled as an increase in doubling time in hours per gray administered) and the number of cell division cycles for which the growth delay factor operates. These modifications made model predictions of growth delay by radiation more consistent with experimental data (Fig 7B and 7C) and provide useful estimates of the magnitude of cell growth inhibition for parameterizing a more mechanistic formulation of radiation damage/repair, integrated into a cell cycle model, planned for future model versions.

### Comparison of simulated and measured PD responses of spheroids to SN30000 and radiation in combination

Given that pharmacologically relevant concentrations of SN30000 (0–150 μM) [38,52] resulted in little cell killing in spheroids under 20% O_2_ with few hypoxic cells (Figs 3B and 5D), spheroids under 5% O_2_ (Figure M) containing similar numbers of both hypoxic and oxygenated cells were used for exploration of SN30000 and radiation combinations. Clonogenic cell survival assays demonstrated that 25 μM SN30000 or 12 Gy radiation alone each killed ca 90% of cells in spheroids under 5% O_2_, while SN30000 in combination with radiation led to almost 99.9% cell kill, showing more than a multiplicative effect. This clonogenic survival data was well predicted by SABM (Fig 8A) using the final parameters from monolayer experiments for the radiation LQ model and reaction-diffusion and cell survival parameters for SN30000 as described above (see Table 1). Model predictions for radiation-induced spheroid growth delay, again incorporating the parameters for cell death probability and cell growth delay (Table 1) by radiation were in good agreement with the experimental results (Fig 8B), although the model under-estimated the growth delay and decrease in size of spheroids in response to the combination.Using the SABM to visualise the plane through the simulated spheroid centre gives insight into the interaction between SN30000 and radiation treatment (Fig 8C). After exposure to SN30000, most hypoxic cells in the inner zone are tagged to die (cells in yellow) while most well-oxygenated cells survive on day 4. These surviving cells are predicted to continue dividing (pink mitotic cells) and to repopulate the spheroid rapidly. The hypoxic cells are predicted to undergo necrosis because of expanding hypoxia as the spheroid grows, even if they are not tagged for death by SN30000, and they make a very limited contribution to spheroid growth because they proliferate slowly. All these factors explain why SN30000 alone results in minimal spheroid growth delay. In contrast, as expected, the SABM predicted that radiation alone preferentially targeted the well-oxygenated, highly-proliferative cells at the periphery of spheroid, sparing some hypoxic cells. The rapid reoxygenation of cells predicted by the SABM results in the rescue of surviving hypoxic cells that would otherwise have died from chronic hypoxia exposure. SN30000 suppresses the hypoxic subpopulation that would otherwise be rescued by this reoxygenation. Overall, the combination of SN30000 and radiation resulted in spatial complementarity by targeting both oxygenated and hypoxic cells in spheroids, therefore substantially delaying spheroid growth. However, the unexpected finding of reduced exposure of SN30000 in the central cells (Figure N) points to a potential limitation of this HAP.

## Discussion

The increasing accessibility of high performance computing is opening up important opportunities for agent-based modelling of large tumour cell populations, with explicit representation of microregional concentration gradients within the tumour microenvironment. At the present time, multiscale modelling of whole tumours with meaningful parameterisation of physiological and pharmacological processes is difficult, but multicellular spheroids provide an experimental model of intermediate complexity that is tractable for such approaches [24,25,28,53]. Here, we report an agent-based model that simulates the growth and response to treatment of tumour spheroids, in which cell fate is determined by local concentrations of oxygen, D-glucose, and exposure to therapeutic agents (SN30000 and radiation). Instead of estimating parameters empirically by fitting spheroids growth data or from the literature, many pivotal parameters were directly measured by experiments in HCT116 cells in a range of different contexts amenable to quantifying transport, metabolism and cytotoxicity (single cell suspensions, monolayers, multicellular layer cultures). This was facilitated by the co-development of a monolayer model, in which all cells have an identical local environment, to estimate the relevant parameters.

The SABM has several invaluable advantages. Firstly, it can temporally and spatially simulate specific cell populations within spheroids. In addition multiple outputs from cellular to histological levels are available from the model, most of which can be directly compared with experimental data. The on-lattice model allowed diffusion to change locally depending on whether a cell is present, and the simple oxygen and glucose metabolism and cell growth models allowed calibration by minimising the number of parameters. Use of predicted radiation and SN30000 response in spheroids allowed detailed exploration and interpretation of radiation and SN30000 combinations. Finally, modelling the total system including the culture medium surrounding the spheroid proved informative. For example, additional validation was possible through simulation of glucose concentrations in the medium, and the predicted decreasing concentration of oxygen in the medium and at the spheroid boundary allowed prediction of the decrease in viable rim thickness (Fig 4) in addition to the geometric change in moving from spherical to planar geometry, resulting in shorter penetration distances in large spheroids caused by spheroid growth and onset of necrosis [48].

Some features and advantages of the SABM are in common with the approach adopted by Powathil et al. [26–28,30], but there are some significant differences. While the Powathil models (PMs) simulate a 2D slice of a real tumour, our model simulates a complete 3D tumour spheroid growing in a tissue culture plate, and as a consequence the boundary conditions are very different. The PMs assume that oxygen is the only constraint on growth *in vivo*, but to simulate spheroid growth *in vitro* it is necessary to account also for glucose depletion in the medium. In the PMs cells are effectively either growing at the unconstrained rate or, when oxygen is low, resting. In contrast, the SABM simulates growth rates that depend on oxygen (and glucose) in a continuous way. Instead of assuming, like the PMs, that cell division can occur only if a neighbouring lattice site is vacant, in the SABM space is made for cell division by moving cells and causing the spheroid to grow. Needing to simulate cell cycle to account for phase-dependent effects, the PMs employ a system of six ODEs, but in the SABM there is no model for cell cycle, time to divide depending simply on growth rate, a function of oxygen and glucose concentrations. In the PMs, drugs are not metabolised and the probability of cell kill in a time step is a fixed value when the drug concentration exceeds a threshold, while our models implement both drug metabolism in cells and kill probability as a continuous function of intracellular drug and oxygen concentrations. This also contrasts with a previous 2D off-lattice model of [41,42] where O_2_ and tirapazamine are simulated by a continuous 2D distribution whereas the PD effect is based on dividing the tumour into 5 discrete hypoxic regions.

Good agreement was found between simulated and measured spheroid growth with the present SABM. This was achieved by adjusting three parameters: a critical oxygen level for cell survival; the time below this critical concentration after which cells are tagged for death; the interval following tagging when cells are still present before undergoing cytolysis. Simulated spheroid diameters, cell number, necrosis and viable rim thickness were compared with measured values and generally agreed well. However, there were some discrepancies between simulation and measurement especially beyond 800 μm diameter. One possible reason is the assumption of symmetry in calculating spheroid diameters for comparison to the simulated spheroids which are very symmetrical compared with the experimental spheroids (Fig 3). In addition, the central necrotic region clearly expanded with spheroid size, and we have observed that HCT116 spheroids can burst randomly at diameters over 1000 μm. All these factors make cell fate and microenvironments in large spheroids complicated and difficult to predict.

Glucose metabolism plays an important role in cancer development and progression [54] and therefore was included in the model. To simplify the models, we assumed that D-glucose was the sole energy and mass resource and that glucose metabolism rate is oxygen independent, although both glycolysis and oxidative phosphorylation are known to be modulated by hypoxia in some cancer cells [55], including a small increase in glucose consumption by HCT116 under chronic hypoxia [56]. Here, we used HCT116 MCL experiments to estimate the diffusion coefficient and metabolism rate of glucose. To our knowledge, this is the first time diffusion of glucose has been directly measured in 3D cell cultures. The estimated diffusion coefficient of ^3^H-L-glucose in HCT116 MCLs was very similar to that of mannitol ((1.81 ± 0.01) ×10^−7^ cm^2^ s^-1^, n = 6), a cell-excluded monosaccharide of similar MW, as expected given that L-glucose is not a substrate for glucose transporters. Assuming that this describes D-glucose diffusion in the extracellular space, a tissue V_max_ was then fitted to the D-glucose transport data, which was in good agreement with the V_max_ fitted (using MABM) to the single cell D-glucose consumption data assuming a tissue-like cell volume fraction of 0.5. Using these parameters, the SABM well predicted D-glucose consumption by spheroids in medium, and spheroid growth in unfed spheroids (Figure I). The low diffusion coefficient and high rate of glucose consumption results in a steep fall of glucose concentration within the spheroid (Fig 2F), which may also affect viable rim thickness. For the other experiments reported here, spheroids were all fed by partial replacement of the medium every second day to ensure sufficient nutrient supplementation. This maintains glucose concentrations an order of magnitude higher than O_2_ and hence it is predicted to be less readily depleted from the medium near the spheroid boundary and under these conditions glucose was found to have little effect of cell growth or cell death with parameters set as in Table 1. However, O_2_ and glucose have similar penetration distance (ca 120 μm) indicating that the hypoxic cells are also glucose deficient, raising the question of control of viability and necrosis. We are currently investigating cell cycling and death under these conditions and developing a model in which glucose metabolism is affected by hypoxia and cell death is related to energy production to address this issue as has previously been done in continuum models [57].

Importantly for our current purposes, the SABM predicted oxygen dependence of clonogenic survival of cells in spheroids by radiation and SN30000, both alone and in combination, without further adjustment of parameters, supporting the conclusion that the oxygen and SN30000 reaction-diffusion parameters and the SN30000 and radiation cell killing parameters accurately describe the distribution of cell killing in spheroids. However, an important objective of this study was to investigate the relationship between clonogenic cell kill and spheroid growth delay, which is a commonly used endpoint in therapeutic testing studies. The SABM under-estimated spheroid growth delay by the combination of SN30000 and radiation. Compared with the clonogenic survival assay (the gold standard endpoint for the determination of radiation-induced cell death), spheroid growth delay induced by radiation is a more complicated PD endpoint that is sensitive to reversible cell cycle arrest, kinetics of cell death and changes in the microenvironment such as reoxygenation after treatment. Metabolic reduction of SN30000 also induces DNA double strand breaks [58,59] and therefore is also expected to cause cell cycle delay. We have developed an empirical model for radiation where cells are assigned a probability of death at each attempted division, similar to that of [24], and that their growth may be slowed for a variable number of cycles. We are investigating a more rigorous formulation treating cell cycle perturbation by radiation and SN30000 explicitly to incorporate into the models. In this approach cell response is dependent on cell-cycle-stage specific radiation- and drug-induced lesion density and repair, which in turn is dependent on dose, and oxygen, rather than the purely probabilistic approach currently implemented.

In the SABM, cell division is the only factor determining cell motion. This is not an issue for untreated spheroids since all lattice sites within the spheroid are occupied by cells except in the central necrotic region. However, it is challenging to quantitate spheroid diameter after treatment. Specifically, after a dose of drug or radiation, surviving cells remain where they were before the dose, leaving many empty lattice sites. This explains why the SABM is unable to predict the spheroid shrinkage in response to radiation and SN30000 that is observed experimentally. To overcome such problems, and generally improve the model’s realism, we are currently developing an off-lattice model in which cell-cell interactions will cause shrinkage of the spheroid when cells undergo cytolysis.

## Methods and materials

### Development of the SABM

Because this model comprises many interacting components, requiring extended explanation, most of the details, including mathematical equations, are presented in the Supplementary document.

The geometry of the model is lattice-based. This means that cells occupy positions, called lattice sites, on a regular 3D grid. A site is the centre of a cube of side Δ*x*, and the grid spacing Δ*x* is set such that in a region of the lattice that is fully occupied by cells (no voids) the fraction of the volume that is taken up by cells agrees with that typically measured for a spheroid constituted of these cells. If the measured average of number of cells per mm^3^ is *N*_*a*_, then this is the number of lattice sites per mm^3^, giving Δ*x* = 1/*N*_*a*_^1/3^ mm.

Autonomous cell motility and cell-cell forces are not simulated. Cell motion occurs only as a result of cell proliferation, but according to rules by which space is made for the newly-created cells, not through solving for a balance of forces. (The method is described in detail in the Supplement.)

The cells that make up the tumour spheroid grow in a specified volume of medium, containing glucose and dissolved oxygen. The oxygen concentration at the medium-air boundary is determined by the gas environment above the medium, and in the present context is constant, but as the spheroid grows and the total oxygen consumption increases, the field of oxygen concentration in the medium constantly changes, and the concentration at the spheroid boundary falls. The concentration of glucose also falls as consumption by growing cells depletes the medium. Concentration fields of the medium constituents are computed by solving the unsteady diffusion equation on a grid using a finite difference method, with the spheroid modelled as a sink of oxygen and glucose. Oxygen and glucose are transported by diffusion into the interior of the spheroid, where they are taken up by cells. At a lattice site (i.e. in a cube of volume Δ*x*^3^) that is occupied by a cell there are two compartments, intra- and extracellular. Diffusive transport is modelled as occurring between the extracellular compartments, while exchanges take place between each intracellular compartment and the extracellular volume that surrounds a cell. The combination of diffusion and intracellular metabolism results in a system of reaction-diffusion equations, expressed using the Method of Lines as a system of ordinary differential equations (ODEs). A parallelised Runge-Kutta algorithm is employed to solve these equations, updating intra- and extracellular concentrations of the constituents at each time step.

The model employs two grids (Figure C): a coarse grid that is used to represent the medium, and a fine grid that defines the lattice sites that determine the domain within which cells can exist. Different methods of solution are used for the two grids. Medium concentrations on the coarse grid are updated in response to cellular fluxes, which are aggregated at nodes of the coarse grid. Interpolation on the coarse grid then provides the concentrations at the boundary of the growing spheroid, thereby providing boundary conditions for the solution–for intra- and extra-cellular concentrations–within the spheroid.

Cell growth is determined by the local glucose and oxygen concentrations. When neither nutrient is limiting the cells grow at a maximum rate calculated from their unconstrained doubling time as in input parameter. Cell growth rate declines with falling oxygen and glucose concentrations with a *K*_*m*_ (concentration for half maximal growth rate) the same as their respective metabolism *K*_*m*_. The mean cell volume is an input parameter and a cell divides into 2 daughter cells when its volume reaches that required for division (as explained in the Supplementary document.)

When growth is simulated in the absence of perturbation by therapeutic agents there are only two constituents, oxygen and glucose, but one of the main purposes of the model is to simulate what happens when drugs are added to the medium. In the most general case there will be six more constituents–two drugs each with up to two metabolites. For both the hypoxia-activated drugs that we are interested in and radiotherapy, treatment effectiveness is greatly influenced by oxygen–in opposite ways. In each case the probability of cell killing is a function of intracellular oxygen concentration. Details of the sub-model for drug metabolism and action, and of the LQ radiation treatment sub-model, are provided in the Supplementary document.

#### Monolayer agent-based model

We also developed a monolayer model (MABM) that includes some features of the spheroid model. In the MABM a relatively large number of cells (of the order of 10^5^) grow, divide and die in a monolayer at the bottom the well. The geometry allows the medium to be treated as one-dimensional, with all cells experiencing the same ambient concentrations of oxygen, glucose and drugs. Since the simple metabolism model does not have cell-cycle stage dependence, although at any instant cells are at different points in the cell cycle, they are all consuming oxygen and glucose and metabolising drugs at the same rate. This enables the simplification of solving the intracellular reactions for a single representative cell, then multiplying by the current number of cells to obtain the total constituent fluxes. More detail of the MABM is provided in the Supplement. The great advantage of this model is its speed of execution.

To facilitate estimation of parameters for the monolayer model, a simple fitting procedure has been developed, using a ‘grid-search’ method outlined in the Supplement. Cellular parameters (such as glucose and SN30000 consumption and SN30000 and radiation survival fractions) were fitted to the experimental data using the monolayer program by minimising the sum of squares of the errors and then used as fixed parameters in the monolayer growth and spheroid ABM simulations for comparison to experiments, as indicated in results.

### Experimental

#### Drugs and reagents

SN30000 was synthesised as reported [60]. SN30000 powder was stored at—20°C and stock solution were dissolved in dimethyl sulfoxide (DMSO) and stored at—80°C. Final DMSO concentrations in cultures were ≤1%.

### Cell culture

The human colorectal adenocarcinoma cell line HCT116 from American Type Culture Collection (Manassas, VA) was cultured as monolayers in alpha minimum essential media (αMEM) (Gibco, Thermo Fisher Scientific, U.S.) with 5% heat-inactivated foetal calf serum (FCS) (Moregate Biotech, Hamilton, New Zealand). The cell line was authenticated by short tandem repeat profiling. Cells were used within 12 passages from frozen stocks in liquid nitrogen. To dissociate cells, log-phase monolayers were exposed to 0.025% trypsin/EDTA (Gibco, Thermo Fisher Scientific, U.S.) for 2 min in an incubator at 37°C, humidified with atmosphere containing 20% O_2_/5% CO_2_ (standard incubator), or spheroids were treated with 0.05% trypsin/EDTA for 10 min in standard incubator. For stirred single cell suspension experiments, cells were collected from enzymatically dissociated multicellular spheroids grown in spinner flasks using 0.05% trypsin/EDTA, with magnetic stirring at 37°C for 10 min. Cells were centrifuged at 216 × g for 5 min and pellets were re-suspended in αMEM without FCS.

#### Oxygen consumption by HCT116 cells

Oxygen consumption rates were determined using a Seahorse XFe96 Analyser (Agilent, U.S.). Cells were dissociated from log phase cultures (αMEM plus 5% FCS) and seeded at 2×10^4^ cells/well in Seahorse 96 well plates and allowed to attach overnight (ca 18 hr). Medium was then changed to Seahorse medium containing 5 mM glucose (150 μL/well). Plates were equilibrated in CO_2_-free 21% O_2_ for 1 hr followed by 3–5 measurements of the oxygen consumption rate at 6 min intervals.

#### Measurement of D-glucose

D-glucose concentrations in the samples were measured with an Amplex Red Glucose/Glucose Oxidase Assay Kit (Invitrogen, CA) according to the manufacturer’s recommendation. Briefly, samples were diluted into reaction buffer to produce D-glucose concentrations of 0 to 50 μM and diluted samples (50 μL) were mixed in a 96-well plate with 50 μL of the working solution containing 100 μM Amplex Red reagent (10-acetyl-3, 7-dihydroxyphenoxazine), 0.2 U/mL horseradish peroxidise and 0.2 U/mL glucose oxidase, followed by incubation at room temperature for 30 min before measuring the fluorescence after mixing using an Enspire Multimode Plate Reader (Perkin Elmer Inc., U.S.) with excitation wavelength of 571 nm and emission wavelength at 585 nm. D-glucose concentrations were determined using a calibration curve (0–100 μM D-glucose) on the same plate.

#### Spheroid culture and growth

10^3^ HCT116 cells in 20 μL αMEM with 10% FCS, 1% P/S were seeded in wells of Corning 7007 low attachment round bottom 96-well plates (Sigma-Aldrich, U.S.) to form spheroids in a standard incubator. After 24 hr, each well was supplemented with 180 μL αMEM with 10% FCS, 1% P/S in a standard incubator for a further 3 days. Spheroids were cultured with replacement of 100 μL αMEM with 10% FCS, 1% P/S and diameters were measured with an ocular micrometer under a microscope (Micro Instruments NZ Ltd, New Zealand) every second day from day 4. In all spheroid experiments, the seeding density (10^3^ HCT116 cells) and spheroid feeding method were the same unless stated specifically. In glucose dependence of spheroid growth experiments, 2 × 10^3^ HCT116 cells were seeded and cultured in glucose-free Dulbecco’s Modified Eagle Medium (glucose-free DMEM) (Gibco, Life Technologies Inc., U.S.) supplemented with 10% FCS, 1% P/S and 5mM D-glucose.

#### Flow cytometry

HCT116 spheroids in 100 μL αMEM with 10% FCS, 1% P/S were exposed for 2 hr to either 100 μM EdU (Abcam, U.S.) to label S-phase cells or 100 μM EF5 (a gift from the National Cancer Institute, U.S.) to label hypoxic cells. 48 spheroids were pooled, trypsinised and cells were collected. For viability assays, cells were incubated in 500 μL PBS supplemented with 1 μg/mL PI for 2 min before counting viable (PI negative) and non-viable (PI positive) cells with an Accuri C6 flow cytometer (B.D. Biosciences, U.S.) using excitation wavelength 488 nm and emission wavelength 585/40 nm. For measurement of hypoxic and S-phase cells, cells were fixed in 1 mL 4% paraformaldehyde (PFA) at 4°C overnight, samples were then diluted to 1% PFA using PBS and stored at 4°C until analysis. For quantitating S-phase cells, EdU in DNA was conjugated with 10 μM Alexa fluor 488 azide (Thermo Fisher Scientific, U.S.) for 30 min using click chemistry according to the manufacturer’s instructions. For measuring hypoxic cells, the cell suspensions were re-suspended in PBStt (PBS with 0.3% v/v tween 20, 0.04g/mL thiomersal and 0.125 g/mL sodium azide) with 75 μg/mL FITC-conjugated Elk 3.52 anti-EF5 antibody (Dr. C. Koch, University of Pennsylvania, Philadelphia, PA) and incubated at 4°C overnight as previously reported [61]. EF5-positive and EdU-positive populations were counted with an Accuri C6 flow cytometer using excitation wavelength 488 nm and emission wavelength 525 nm.

#### Histological staining of spheroid sections

Following exposure to EdU or EF5 as described above, spheroids were washed with PBS three times, pooled in 1 mL micro-centrifuge tubes, and then fixed with 4% PFA at 4°C overnight before being dehydrated in 70% v/v histological ethanol. The spheroids were then embedded in paraffin for sectioning. The sections were deparaffinised and dehydrated by sequential washes in xylol, ethanol and distilled water. For EdU staining, sections were permeabilised with PBStt for 30 min at room temperature, followed by exposure to click reaction cocktails with 10 μM Alexa fluor 647 azide (Thermo Fisher Scientific, U.S.) at room temperature for 30 min. For EF5 staining, antigen retrieval was performed using 10 mM citrate buffer (pH = 6.5) in a 2100 Retriever (Electron Microscopy Sciences, U.S.), followed by blocking in Tris buffered saline/0.1% Tween-20 (TBS-T) containing 10% goat serum at 4°C for 1 hr. The sections were then submerged under 100 μL antibody mixture containing 75 μg/mL CY5 conjugated Elk 3.52 anti-EF5 antibody at 4°C overnight. Slides were then counterstained with 100 μL of 8 μM Hoechst 33342 (Thermo Fisher Scientific, U.S.) for 10 min before mounting with ProLong Diamond Antifade Mountant (Invitrogen, U.S.). For H&E staining, coverslips were removed after imaging EdU/EF5, and the slides were stained with haematoxylin and eosin and then mounted with DPX Mountant (Sigma-Aldrich, U.S.). The images were taken on a Zeiss LSM 710 inverted confocal microscope.

#### Diffusion and metabolism of SN30000 and glucose in HCT116 MCLs

HCT116 MCLs were cultured as described previously [50]. Briefly, 1 x 10^6^ cells were seeded onto collagen-coated Millicell-CM culture inserts (Merck Millipore Ltd., Ireland), allowed to attach for 6 hr then submerged in stirred reservoirs of culture medium at 37°C. After 3 days MCLs were loaded into custom-designed diffusion chambers in a 37°C waterbath and equilibrated with 95% O_2_/5% CO_2_ for 1 hr in αMEM without FCS before adding the test compounds. SN30000 (10–50 μM), D-glucose (5–10 mM), or L-glucose (1 mM) plus 5 μL ^3^H-L-glucose (Glucose, L-[1-^3^H(N)], 740 GBq/mmol, Amersham, Australia) [62] was added to the donor compartment, along with 1 μL ^14^C-urea (2.11 GBq/mmol, Amersham, Australia) as internal standard. 100 μL of medium was sampled from both donor and receiver compartments at intervals. Radioactivity was measured in 25 μL samples by liquid scintillation counting (Tri-Carb 2910 TR, Perkin Elmer Inc., U.S.) to determine ^3^H-L-glucose and ^14^C-urea, the latter to estimate MCL thickness by using the known diffusion coefficient of urea in HCT116 MCLs as previously [50]. The remaining samples were frozen at -80 ^0^C for determination of SN30000 or D-glucose. Diffusion coefficients of the analytes in the MCL were estimated by fitting data to concentrations calculated using a Matlab program to solve the transport model based on Fick’s second law as described previously [50]. Identical experiments were performed under 0% O_2_ in the gas phase for determination of the rate constant for bioreductive metabolism of SN30000 in the MCLs under anoxia, together with measurement of the bioreductive metabolite SN30000-1-oxide. Analogous experiments were performed with collagen-coated support membranes without cells to determine the diffusion coefficient of D-glucose, SN30000 and SN30000-1-oxide in bare supports (*D*_*sup*_). Medium diffusion coefficients were calculated from *D*_*sup*_ by dividing by the previously estimated bare support porosity of 11% [63].

#### Irradiation of monolayers and spheroids

96-well plates with log-phase monolayers (10^5^ cells/well, seeded 3 hr previously) or 4 day old spheroids were sealed in a metal chamber and submerged in a 37°C waterbath for 30 min to equilibrate temperature before being exposed to gamma radiation from an Eldorado 78 Cobalt-60 teletherapy machine. A range of dose rates (0.2–1 Gy/min) was achieved by setting a lead wedge on the top of the metal chamber [64,65]. For radiation exposure experiments under 5% O_2_, HCT116 spheroids in 100 μL αMEM with 10% FCS, 1% P/S were pre-incubated in a Whitley H45 HEPA HypOxystation (Don Whitley Scientific Limited, U.S.) with 5% O_2_/90% N_2_/5% CO_2_ for 3 hours to equilibrate oxygen before being exposed to radiation.

#### Clonogenic cell survival assay

To measure clonogenic survival of HCT116 cells treated by SN30000 or radiation, single cell suspensions from trypsinised monolayers or spheroids were counted (Beckman Coulter Z2, U.S.), serially diluted and plated into 60-mm cell culture dishes (Falcon, U.S.) containing 4.5 mL αMEM with 5% FCS and 1% P/S. Cells were cultured in a standard incubator for 10 days before being stained with methylene blue, and colonies of >50 cells were counted to calculate plating efficiency (PE, number of colonies/cells plated). Surviving fraction (SF) was calculated as PE (treated)/PE (control). For monolayers, surviving fractions were fitted as a function of dose and O_2_, to the LQ radiation model (Eqn S12) to estimate *α*_*H*_, *β*_*H*_ and the oxygen enhancement ratio (assuming *OER*_*α*_ = *OER*_*β*_).

#### High-Performance Liquid Chromatography (HPLC)

SN30000 and its metabolites were quantitated by HPLC with photodiode array detection using a Zorbax Eclipse XDB-C18 column (2.1 x 150 mm, 5 μm particle size, Agilent, U.S.) on an Agilent 1100 HPLC system. Samples of culture medium (without FCS) were thawed, centrifuged and 40 μL samples were injected for analysis. The mobile phase (pH 3.5) comprised 45 mM sodium formate buffer with 0.2% formic acid and the organic phase was 60% acetonitrile, 20% methanol, 20% water. The gradient was 10% organic phase for 2 min with linear increase to 40% (2–16 min) and to 95% at 19 min. Absorbance detection was at 252 nm with a reference of 550 nm. For each experiment, calibration curves for SN30000 and its 1-oxide metabolite were determined under the same sample-handling conditions.

#### Spheroid area measurement

An ImageJ plugin was developed (based on a macro provided by [66]) to estimate the area and roundness metric from the 2D projected bright field spheroid images captured by a JuLi stage Real-Time Cell History Recorder (NanoEnTek Inc., Korea) using a 4× objective. After scaling has been set, the analysis employed the following sequence of ImageJ filter steps: "Auto-thresholding" step using the inter-modes algorithm, a single "Erode" step, "Analyse particles" step to detect objects with area greater than a specified number of pixels, default value 1000. This selects the spheroid, filtering out any surrounding debris, and estimates its area and a roundness metric (in the range 0–1) automatically for a full set of spheroid images.

#### Viable rim and necrotic core measurement

For histology analysis, another plugin was applied manually image-by-image, using ImageJ’s “Magic Wand” to find the ROI of the whole spheroid, then the “Free-hand” tool to delineate the ROI of the necrotic core. In each case the associated area is computed.

From each area the radius of the equivalent circle is determined as $\sqrt{\frac{area}{\pi }}$, giving the viable rim thickness as the difference between the two radii.

## Supporting information

S1 Text Supplementary information including the detailed description of the agent based model and supplementary figures:
Figure A. Flow of program execution, showing the main modules. The loop is executed every time step (typically 600 sec.)
Figure B. 2D representation of lattice sites with and without an occupying cell.
Figure C. Plan view (XZ) showing the lattice grid (red) and part of the coarse grid (blue). For a medium volume of 0.2mL, the coarse grid is 35×35×40, with grid spacing = 164 μm. The lattice grid is 120×120×120, with grid spacing = 12.6 μm.
Figure D. The range of real-time graphical displays of spheroid state available in the GUI. (**a**) time-series plot of number of cells killed by hypoxia, (**b**) profile plot of EC oxygen concentration within the spheroid, (**c**) 2D plot of oxygen concentration in the fine grid, light green cells are oxic, dark green cells are hypoxic (< 1μM O_2_), blue cells are tagged to die, pink cells are in mitosis, black region is necrotic, (**d**) 3D rendition of spheroid, (**e**) Flow cytometry plot of oxygen vs. CFSE (a dye that indicates cell generation), (**f**) log-scale histogram of IC oxygen level.
Figure E. The main GUI results screen, showing 8 of the 32 available plots.
Figure F. HCT116 monolayer growth (a) and glucose consumption (b). The MABM was used to estimate the doubling time, T_d_, based on observation of HCT116 monolayer growth. HCT116 monolayers (5×10^3^ cells/well) in 6-well plates with 4 mL of αMEM supplemented with 10% or 5% FCS were cultured in 20% O_2_/5% CO_2_ humidified incubator without medium replenishment. Cell number and glucose concentrations in specific wells were measured. Lines are model fits to the cell count and glucose concentration data. T_d monolayers_ was the fitted parameter with glucose metabolism parameters fixed at the estimated values in Table 1.
Figure G. Survival of HCT116 cells under anoxia. HCT116 monolayers (2×10^4^ cells) in 6-well plates with 4 mL of αMEM+5% FCS were exposed to anoxia at 37°C (anoxic chamber) for the indicated times before dissociation, counting and plating for clonogenic survival assay. Points are mean ± SEM for 3 replicates.
Figure H. Quantitation of cellular characteristics of HCT116 spheroids by flow cytometry. Representative scatter plots of cell viability (% PI negative), hypoxic fraction (% EF5-positive cells) and S-phase fraction (% EdU-positive cells) for day 3—day 9 spheroids. Summary data are shown in Fig 5.
Figure I. Oxygen dependence and un-fed spheroid growth and comparison with the SABM. (**a)** HCT116 spheroids (seeded with 2×10^3^ cells/well) were cultured under 20%, 5% or 1% O_2_ and the diameters of spheroids were monitored (points) during medium change every 2^nd^ day and simulated (lines) as a function of time. Simulations are based on the model parameters in Table S1. Experimental values are means ± SD for 4 replicates. (**b, c)** HCT116 spheroids (seeded with 10^3^ cells/well) were cultured in glucose-free DMEM with 10% FCS supplemented with an initial concentration of 5 mM D-glucose without replacement of the medium. Spheroid diameter (points in **b**) was measured on the indicated days, as was the concentration of D-glucose in medium (points in **c**). Values are means ± SD for 4 replicates. The SABM simulations, based on model parameters in Table S1 show good agreement with experimentally determined spheroid growth (lines in **b**) and consumption of D-glucose in medium (lines in **c**).
Figure J. SN30000 metabolism by 1-electron reductases and proposed mechanism of cytotoxicity. SN30000 is metabolised by 1-electron reductases (1) to an initial radical which is re-oxidised to SN30000 in the presence of O_2_ (2) providing hypoxic selectivity. The initial radical may undergo further reduction to the 2 electron of 4 electron reduction products (1-oxide and nor oxide, steps 3 & 4) or formation of an oxidising benzotriazinyl radical capable of causing initial DNA damage. These radical anions are short lived and retained within the cell of origin. It is proposed that SN30000, its 1-oxide or oxygen can oxidise the initial DNA radical (7) resulting in strand breaks that then become complex DNA lesions. For more details see [39,58,67]
Figure K. Development of a spatially resolved PK/PD model for SN30000. Supplementary to the data in Fig **6**, bioreductive metabolism of SN30000 under anoxia was confirmed by the appearance of SN30000-1-oxide in medium (**a**) in anoxic stirred single cell suspensions, and in the donor (**b**, filled symbols) and receiver (**b**, open symbols) compartments in MCL experiment for determining SN30000 diffusion with predictions assuming 75% conversion to SN30000-1-oxide. Each MCL in Fig 6 was of similar thickness as estimated from diffusion of ^14^C-urea (**c**).
Figure L. Cell killing by SN30000 in stirred cell suspensions under 20% O_2_ at 2 initial SN30000 concentrations. Lines are model fits using the MABM assuming the medium was fully stirred, that oxygen effects the rate of metabolism according to Eqn (S14) and the same PD model as used under anoxia in Fig 6 (Model 2, Eqn (S19)). The fitted parameter was the Hill coefficient for oxygen dependence of SN30000 metabolism in Eqn (S19).
Figure M. Hypoxic fraction of spheroids under 5% O_2_. The SABM predicted higher hypoxic fraction of spheroids (with diameter ca. 450 μm) pre-incubated under 5% O_2_ for 3 hr (**a**) than that under 20% O_2_ (Fig 3C). To confirm this experimentally, 4 day HCT116 spheroids pre-incubated under 5% O_2_ for 3 hr were then exposed to hypoxia probe EF5 for 2 hr, followed by dissociating spheroids to single cell suspensions for flow cytometry (**b**) or by fixing and sectioning spheroids for immunostaining (**c**).
Figure N. Upper Row: O_2_ (**a**), glucose (**b**) and SN30000 (**c**) concentration profiles under treatment conditions when spheroids (ca. 460 μm) were exposed to SN30000 under 5% O_2_. Lower Row (**d-f**): Predicted concentration profiles for SN30000 exposure under the indicated oxygen conditions are shown for comparison.
(PDF)
