# Supplementary material for: An agent-based model for drug-radiation interactions in the tumour microenvironment: Hypoxia-activated prodrug SN30000 in multicellular tumour spheroids
Source: PLoS Comput Biol. 2018 Oct 24;14(10):e1006469. doi: 10.1371/journal.pcbi.1006469 (PMC6218095; doi:10.1371/journal.pcbi.1006469)
Supplement: S1 Text — Supplementary information including the detailed description of the agent based model and supplementary figures: Figure A. Flow of program execution, showing the main modules. The loop is executed every time step (typically 600 sec.) Figure B. 2D representation of lattice sites with and without an occupying cell. Figure C. Plan view (XZ) showing the lattice grid (red) and part of the coarse grid (blue). For a medium volume of 0.2mL, the coarse grid is 35×35×40, with grid spacing = 164 μm. The lattice grid is 120×120×120, with grid spacing = 12.6 μm. Figure D. The range of real-time graphical displays of spheroid state available in the GUI. (a) time-series plot of number of cells killed by hypoxia, (b) profile plot of EC oxygen concentration within the spheroid, (c) 2D plot of oxygen concentration in the fine grid, light green cells are oxic, dark green cells are hypoxic (< 1μM O2), blue cells are tagged to die, pink cells are in mitosis, black region is necrotic, (d) 3D rendition of spheroid, (e) Flow cytometry plot of oxygen vs. CFSE (a dye that indicates cell generation), (f) log-scale histogram of IC oxygen level. Figure E. The main GUI results screen, showing 8 of the 32 available plots. Figure F. HCT116 monolayer growth (a) and glucose consumption (b). The MABM was used to estimate the doubling time, Td, based on observation of HCT116 monolayer growth. HCT116 monolayers (5×10^3^ cells/well) in 6-well plates with 4 mL of αMEM supplemented with 10% or 5% FCS were cultured in 20% O2/5% CO2 humidified incubator without medium replenishment. Cell number and glucose concentrations in specific wells were measured. Lines are model fits to the cell count and glucose concentration data. Td monolayers was the fitted parameter with glucose metabolism parameters fixed at the estimated values in Table 1. Figure G. Survival of HCT116 cells under anoxia. HCT116 monolayers (2×10^4^ cells) in 6-well plates with 4 mL of αMEM+5% FCS were exposed to anoxia at 37°C (anoxic chamber) for [file pcbi.1006469.s001.pdf]

Mao et al.

An agent-based model for drug-radiation interactions in the tumour microenvironment: hypoxia-activated prodrug SN30000 in multicellular tumour spheroids.

Supporting information

Description of SABM

The overall structure of the model is shown in Figure A. In the following the main model components are described.

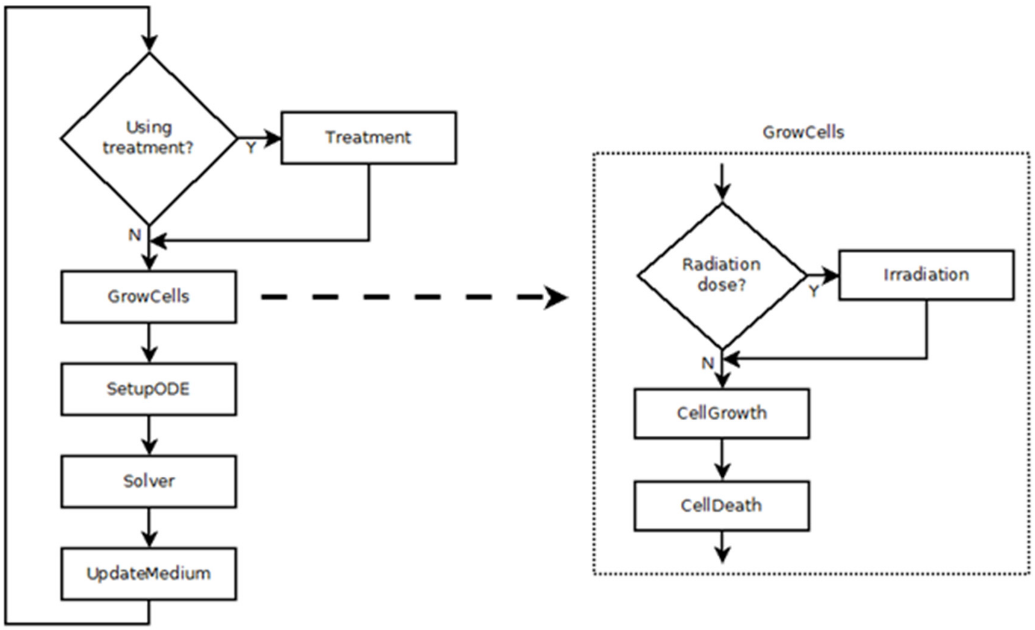

Figure A. Flow of program execution, showing the main modules. The loop is executed every time step (typically 600 sec.)

Reaction-diffusion system

Because the spheroid grows in a small volume of growth medium, it has a significant influence on constituent concentrations in the medium and at the spheroid boundary, causing levels to change constantly, and making it necessary to include the medium in the simulation. The medium is assumed to be unmixed, although since there is always some mixing by convection, the effective diffusion coefficients accounting for such unpreventable mixing need to be estimated. A two-stage solution procedure is used:

Stage 1. The concentration fields throughout the whole volume are updated each time step by solving the time-dependent diffusion equation on a regular rectangular grid. The time-development of these fields is driven by fluxes created by cells in the spheroid. All boundaries are closed except for oxygen, for which the boundary value of concentration at the medium-gas phase interface is an input value corresponding to the experiment being simulated. In the cases of oxygen and glucose, each cell is a sink, while for drugs and their metabolites a cell can be either a sink or a source. Since the coarse grid solver requires flux values at its grid points, a method is needed to transfer fluxes occurring at intermediate points (cell locations) to coarse grid points. At each time step, for each constituent the current cell uptake rates are allocated to the eight surrounding grid points using precomputed weighting factors that are inversely proportional to the distance from the grid point. From the resulting solution the concentration in the medium close to the boundary of the spheroid is estimated by averaging concentrations at 100 randomly chosen points on the surface of a sphere representing the current size and location of the spheroid. Concentrations at these points are determined by trilinear interpolation. (This module is shown as UpdateMedium in the flowchart.)

Stage 2. The medium boundary concentrations determined from Stage 1 are applied to lattice sites at the boundary of the spheroid. (A boundary site in this context is a lattice site outside

the spheroid that has at least one neighbour site that is occupied by a cell.) The lattice solver updates the intra- and extracellular concentrations within the lattice, treating the boundary concentrations as fixed. Cellular constituent fluxes are then computed from the intra- and extracellular concentrations, and used to generate grid point fluxes for the Stage 1 solution in the next time step. (This module is shown as Solver in the flowchart.)

The time step is user-settable in the GUI, and typically the value of 600 seconds is used (this is subdivided on a medium-change event, as discussed below.)

Solving the diffusion equation in the medium (Stage 1)

The medium and spheroid are simulated as occupying an approximately cuboid region with volume – an input value – matching that used experimentally in the 96-well plates. Medium volume is much greater than the maximum volume attained by the spheroid. There is no attempt to reproduce the geometry of a well, which is cylindrical with a slightly concave bottom, but the volume is preserved and the cross-sectional area is approximately preserved, ensuring that the medium depth is close to the experimental value – this is important for oxygen levels. The cube is spanned by a "coarse" regular finite difference grid *NXB*NYB*NZB*, with $NYB = NXB$, and with grid spacing Δx_B that is determined by the volume. For example, when the volume of medium is 0.2 mL, NXB=NYB=35, NZB=40, and Δx_B = 164 μm.

Because drugs may decay in the medium, it is necessary to consider a general reaction-diffusion equation of this form:

$$\frac{\partial C}{\partial t} = F(C) + D\nabla^2 C \qquad \text{(S1)}$$

where C is constituent concentration, D is the diffusion coefficient and F represents the reaction kinetics – in the present case simple first order decay. The solution method adopted is a

linearized multi-step implicit-explicit (IMEX) scheme, specifically the 2nd-order semi-implicit BDF formulation described in [1]:

$$\frac{1}{2\Delta t}\left[3C^{n+1} - 4C^{n} + C^{n-1}\right] = 2F(C^{n}) - F(C^{n-1}) + D\Delta_{h}C^{n+1} \tag{S2}$$

(Here the index n refers to the time point $n\Delta t$.)

The resulting algebraic system is solved using the sparse solver ITSOL, employing FGMRES with ILUK preconditioning [2,3]. OpenMP parallelisation enables solving for all constituents in parallel.

Solving the reaction-diffusion equations on the lattice (Stage 2)

Within the coarse grid a much finer grid is specified (Figure C, below). This is the lattice within which cells are located, and it is sized to accommodate the largest spheroid to be simulated – the default size is 120×120×120. The lattice grid spacing is determined by the number of tumour cells per unit volume in a spheroid (as explained below). For HCT116 cells, the grid spacing Δx is 12.6 µm. At any time the spheroid occupies N_S sites on the 3D lattice. The number of live cells, N_C, may be less than N_S, because the spheroid may have voids as a result of cell death. If there are M_V constituents the total variable count in the spheroid is $M_V(N_S + N_C)$. There are two domains, extracellular and intracellular, and the solver must account for the possibility that a lattice site may be unoccupied by a cell, in which case the whole site volume is extracellular. Figure B shows the two cases, using a 2D representation for clarity.

Extracellular equations: constituents are transported by diffusion through the extracellular domain. In each site containing a cell, there is exchange across the cell membrane. The only other possible reaction in the extracellular space is decay – all other reactions occur within cells.

Intracellular equations: constituents are transported into and out of the cell through exchange across the cell membrane. Oxygen and nutrients are consumed at rates determined by metabolic functions. Drugs are metabolised, and constituents may be subject to decay.

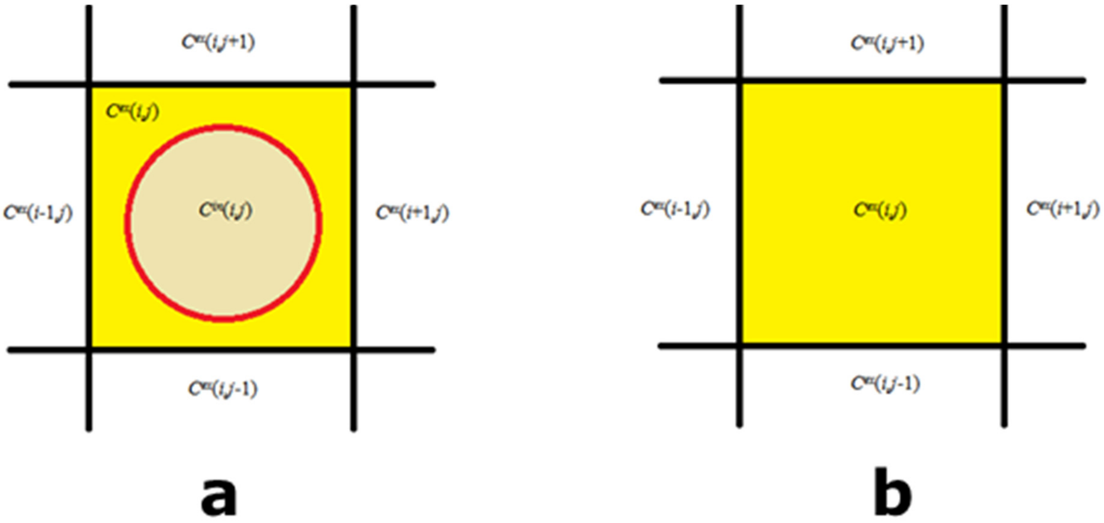

Figure B. 2D representation of lattice sites with and without an occupying cell.

Using the Method of Lines, the diffusion PDE is converted to an equivalent set of ODEs. In the case of a vacant site there is simply one ODE for the extracellular concentration (for clarity, dropping the '*ex*' superscript) at site (*i*,*j*,*k*):

$$L_1 = \left(\frac{D}{\Delta x^2}\right)\left(C_{i-1jk} + C_{i+1jk} + C_{ij-1k} + C_{ij+1k} + C_{ijk-1} + C_{ijk+1} - 6C_{ijk}\right) \tag{S3}$$

$$\frac{dC_{ijk}}{dt} = L_1 - K_{decay}\, C_{ijk} \tag{S4}$$

If the site is occupied by a cell, there is one ODE for the extracellular compartment and one for the intracellular:

$$L_2 = \left(\frac{\Delta x\, D}{V_{ex}} \right) \left(C_{i-1jk} + C_{i+1jk} + C_{ij-1k} + C_{ij+1k} + C_{ijk-1} + C_{ijk+1} - 6C_{ijk} \right) \tag{S5}$$

$$\frac{dC_{ijk}}{dt} = L_2 - K_{decay} C_{ijk} - \frac{U'(C_{ijk}, C_{ijk}^{in}, V_{in})}{V_{ex}} \tag{S6}$$

$$\frac{dC_{ijk}^{in}}{dt} = \frac{U'(C_{ijk}, C_{ijk}^{in}, V_{in}) - M'(C_{ijk}^{in})}{V_{in}} - K_{decay} C_{ijk}^{in} \tag{S7}$$

V_{in} is the cell volume, and V_{ex} is the extracellular volume = $\Delta x^3 - V_{in}$, in units of cm^3.

In the simplest formulation, the mass rate of uptake U' depends on the concentration difference across the cell membrane and the cell surface area. In reality the rates of diffusion into and out of the cell can depend on the weighted concentration difference, therefore the model allows specification of two membrane mass transfer coefficients to characterize the uptake rate: K_{in} and K_{out}.

$$U'(C_{ijk}, C_{ijk}^{in}, V_{in}) \propto (K_{in} C_{ijk} - K_{out} C_{ijk}^{in}) V_{in}^{2/3} \tag{S8}$$

For each constituent there is an equation for the molar rate of metabolism M'. In the case of drugs and their metabolites the rate of consumption (or production) depends in general on the oxygen concentration and concentrations of related drug constituents - the drug reaction equations are discussed below. (Note that for drug metabolites the molar rate of metabolism M' is the difference between the consumption rate and the rate at which the metabolite is produced.) The molar rates of consumption of oxygen and glucose are given by Hill functions:

$$M'(C) = \frac{C^n}{C^n + C_0^n} r_{\max} \tag{S9}$$

n and C_0 are Hill and Michaelis-Menten parameters, r_{max} = maximum consumption rate per cell in μmol.s^{-1}. These parameters are specified in the input file. The rates of metabolism of nutrients drive cell growth; this is treated explicitly, and cell growth is solved separately from the reaction-diffusion system. Cell growth, division and death are discussed below.

Method of solution:

Because the number and distribution of cells in the spheroid are constantly changing, the system of equations must be reassembled every time step. To reduce the run time, and thereby enable simulations with a large number of cells, parallelisation with OpenMP is employed. In each time step the equations for each constituent are solved for all intra- and extracellular concentrations, holding the concentrations of the other constituents fixed. Glucose concentrations are updated, then the drug concentrations (if drugs are present), then oxygen concentrations. The variable time step Runga-Kutta-Chebychev solver [4,5] that is used for all constituents except oxygen makes repeated calls to a parallelised procedure that computes the rate of change of the concentration vector.

In the case of oxygen a different approach is adopted. Because oxygen equilibrates very rapidly, it is appropriate to assume that the oxygen concentration field within the lattice is always at steady state for the current spheroid boundary conditions. A successive under-over-relaxation method is used to find the equilibrium solution. An important assumption in this method is that because intracellular processes involving oxygen are so fast, effectively the intracellular oxygen concentration is always in equilibrium with the extracellular value. This means that the oxygen consumption rate matches the rate of transport across the cell membrane, which makes it possible to calculate the intracellular concentration from the extracellular concentration. If the Hill exponent is 1 a quadratic is solved, if it is 2 a cubic is solved. Eqtn. (8) is then used to obtain the oxygen uptake rate.

The under-over-relaxation procedure (also parallelised with OpenMP):

The current vector of extracellular concentrations provides the initial guess $\mathbf{y}_0$

$\mathbf{y}_1 = \mathbf{y}_0$

under-relaxation loop start:

 uptake rate vector U' determined from $\mathbf{y}0$

 over-relaxation loop start:

 eqns (3)-(7) => C for all spheroid sites, with $dC/dt=0$, U' fixed

 $\mathbf{y}_1 = (1-w_{\text{over}}).\mathbf{y}_1 + w_{\text{over}}\boldsymbol{C}$

 exit loop on convergence

 over-relaxation loop end

 $\mathbf{y}_0 = (1-w_{\text{under}}).\mathbf{y}_0 + w_{\text{under}}\mathbf{y}_1$

 exit loop on convergence

under-relaxation loop end

$\mathbf{y}_0$ is the updated solution

The convergence criterion in each case is that the fractional change in all concentrations is less than the supplied tolerance (1.0e-6). The relaxation factors used are: $w_{\text{over}} = 1.6$, $w_{\text{under}} = 0.05$. When the vector of extracellular concentrations has been updated, intracellular concentrations are deduced from $\mathbf{y}_0$.

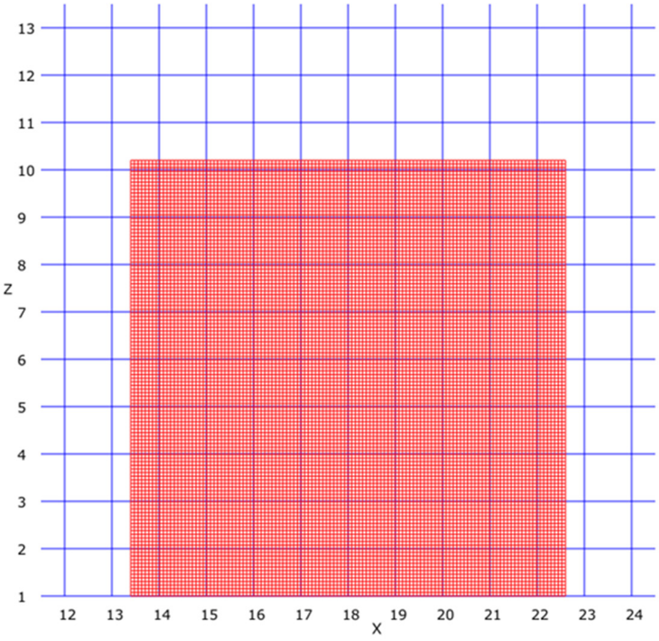

Figure C. Plan view (XZ) showing the lattice grid (red) and part of the coarse grid (blue). For a medium volume of 0.2mL, the coarse grid is 35×35×40, with grid spacing = 164 µm. The lattice grid is 120×120×120, with grid spacing = 12.6 µm.

Cell growth, division and death

Growth

Currently the model does not simulate cell cycle stages explicitly. Instead cell volume is used as the index of growth. The rate of volume increase is proportional to the metabolic rate, which in turn is proportional to the product of the rates of metabolism of oxygen and glucose, each of which is represented by a Hill function, as mentioned above.

$$\frac{dV}{dt} = \frac{C_{O2}^{n1}}{C_{O2}^{n1} + C_{0\_O2}^{n1}} \frac{C_g^{n2}}{C_g^{n2} + C_{0\_g}^{n2}} r_{\max} \tag{S10}$$

where C_{O2} is the intracellular oxygen concentration, C_g is the intracellular glucose concentration, and $n1$, $n2$, C_{0_O2} and C_{0_g} are the respective Michaelis-Menten parameters for oxygen and glucose. The change in cell volume V in the time step is $\frac{dV}{dt}\Delta t$.

A cell grows until its volume reaches a predetermined threshold for division, V_{div}. An input parameter f_{div} (default value 1.6) sets the volume at division through $V_{div} = f_{div}V_n$, where V_n is the nominal cell volume. The nominal cell volume V_n and the lattice grid spacing Δx are determined together from f_{div} and two supplied parameters:

N = # of cells/mm^3 = # of lattice sites/mm^3 (since there is one cell per lattice site)

ff = fraction of spheroid that is fluid.

If Δx is in units of μm, $N\Delta x^3 = 10^9$, $\Delta x = 1000N^{-1/3}$. For HCT116 cells, N = 500,000 cells/mm^3, which gives Δx = 12.6 μm. The volume of a cubic lattice site is $V_{site} = \Delta x^3$ μm^3 = 2 pL. A cell divides when volume = $f_{div}V_n$, to yield two cells with volume $f_{div}V_n/2$. Therefore in a well-oxygenated growing spheroid the average cell volume is $V_{ave} = 0.75f_{div}V_n$ (assuming that cell size is approximately uniformly distributed.) If ff = fraction of spheroid that is fluid, 1-ff = fraction that is cells. Therefore $1\text{-}ff = V_{ave}/V_{site}$, or $0.75f_{div}V_n = (1\text{-}ff)V_{site}$, yielding $V_n = (1\text{-}ff)V_{site}/(0.75f_{div})$. For HCT116 cells ff = 0.5, therefore f_{div} = 1.6 gives V_n = 0.8333 pL, V_{ave} = 1.0 pL.

Since post-division each progeny cell has volume = $V_{div}/2$, the change in volume over the cell cycle is $V_{div}/2$. For a well-nourished cell (with no nutrient constraints) the duration of the cell cycle is therefore $V_{div}/(2r_{max})$. The cycle time for a given cell line, T_{div}, is an input parameter, and $r_{max} = V_{div}/(2T_{div})$. (To prevent all well-nourished cells having exactly the same divide time, variability in cycle time is introduced by randomising V_{div} by adding an increment dV uniformly distributed in a small range about zero, typically -0.15 – 0.15.)

When the cell growth is computed, and cell volume increases by ΔV, an adjustment is necessary to ensure mass conservation of the various constituents. This necessity arises because in the lattice formulation the intra- and extracellular volumes sum to the fixed site volume V_{site}, therefore an increase in cell volume of ΔV implies a decrease in extracellular volume of ΔV, but these equal volumes have different concentrations. Without some adjustment, the total constituent masses would change. Clearly many adjustment procedures are possible. The method adopted is to assume that a transfer of ΔV at extracellular concentrations takes place, automatically ensuring mass conservation. In other words, the extracellular concentrations are unchanged, but the intracellular concentrations are modified. If the cell volume and intracellular concentration vector before growth are V_{in} and C_{in}, and the extracellular volume and concentration vector are $V_{ex}=V_{site} - V_{in}$ and C_{ex}, then the adjusted intracellular concentration of a constituent is:

$$C = \frac{V_{in}C_{in} + \Delta V C_{ex}}{V_{in} + \Delta V} \tag{S11}$$

Death by oxygen or glucose deprivation

Cells that suffer prolonged deprivation of either oxygen or nutrients (represented here by glucose) eventually die. This is modelled as a two-stage process. Focussing on oxygen (the same procedure applies with glucose), when the intracellular level falls below the specified threshold ($\text{Tag-conc}_{\text{oxygen}}$, Table 1) for anoxia a timer is started. If the level stays below the threshold for a specified duration ($\text{Tag-time}_{\text{oxygen}}$, estimated value 24 hr, Table 1) the cell is tagged to die of anoxia. If the oxygen level rises above the anoxia threshold before the cell is tagged it survives and continues to grow as usual. A tagged cell does not die immediately, instead there is a specified delay ($\text{Death-delay}_{\text{oxygen}}$, estimated value 24 hr, Table 1) before death

occurs and lattice site occupied by the cell is set to unoccupied, and the cell is removed from the simulated list.

Division

A cell enters division when its volume reaches the predetermined threshold for division. At this point if the cell has been tagged to die from radiation or drug exposure (see the section on Treatment below), it dies instead of dividing thus simulating post-mitotic cell death.

When a cell divides each progeny cell has an initial volume exactly half that of the parent, and concentrations of all intracellular constituents are unchanged. One of the progeny cells remains on the site of the parent cell (S_0), and a suitable site must be chosen for the other cell. If there is a vacant site that is inside the spheroid and adjacent to S_0 then this is used (i.e. it is S_{01} in Step 4 below; no need for Steps 1-3). In the event that there is more than one vacant site, the site with the highest oxygen concentration is chosen. If there are no vacant sites, it becomes necessary to create a vacancy adjacent to S_0 by moving cells. The multi-step procedure by which this is achieved is now described.

Step 1. One of the neighbour sites of S_0 is chosen randomly – call this S_{01}. If S_{01} is outside the spheroid no movement of cells is needed. Otherwise a boundary site S_B is selected to be the end of the path through the lattice along which cells will be moved to create space at S_{01}. A boundary site is inside the spheroid and has at least one Neumann neighbour that is outside the spheroid. Dual criteria are used to select S_B. The site should be near S_{01}, for obvious reasons, but since the cell currently at S_B will be moved to a site that is currently outside the spheroid, the selection has implications for the way the shape of the spheroid changes. In fact it is the algorithm used to select S_B that ensures that the spheroid maintains a roughly spheroidal shape.

Rather than choose the closest boundary site, the approach used is to search within a nearby region of the spheroid surface for the boundary site that departs most from the desired spherical shape, in a specific sense. If the spheroid centre is at S_C, the search region is defined by boundary sites S such that the angle between vectors $S–S_C$ and $S_{01}–S_C$ is less than a threshold value $\alpha_{max}=f(r)$, a function of the fractional distance from S_{01} to the spheroid centre: $r = |S_{01}-S_C|/R_S$, where R_S is the current spheroid radius. The idea is that the closer S_{01} is to the spheroid surface, the more the search is focused on sites near S_{01}. The way α_{max} depends on r is determined by three parameters: $r_m = 0.8$, $\alpha_1 = \pi/2$, $\alpha_2 = \pi/6$ as follows:

If $r < r_m$ then $\alpha_{max} = (1 - r/r_m)\ \alpha_1 + (r/r_m)\ \alpha_2$, else $\alpha_{max} = \alpha_2$.

In the search region S_B is chosen as the site closest to S_C, in other words a location where the departure from the desired sphere is likely to be an indentation to be filled.

Step 2. The most direct path through the lattice from S_{01} to S_B is found. The path is determined as a sequence of jumps from a site to one of its 26 neighbour sites, starting at S_{01}. The optimal jump from a site minimizes the distance to S_B. Because cell death creates voids in the spheroid, it is possible that a neighbour site may be vacant. When this occurs the path is terminated before it reaches the spheroid boundary.

Step 3. Starting at the end of the path (either the boundary site S_B or the site before the interior vacant site) cells at sites along the path are moved in sequence, culminating in the move that frees up site S_{01}.

Step 4. The second progeny cell is placed in S_{01}.

Treatment

The treatment protocol is read from the input file when the run starts, and stored as a sequence of timed events. The treatment events are of three kinds: radiation dose, drug dose, and medium

replenishment. Medium replenishment simulates the normal experimental procedure for resupplying glucose. In a drug dose event the medium is changed twice: when the drug is administered, and when the medium is replaced at the end of the dosing period. The dose event is described by five values: the volume of medium (containing drug) exchanged, the concentration of drug in the added medium, the exposure time, i.e. the time interval before the medium is again replenished, the oxygen concentration while the drug is present, and the oxygen concentration in the added medium. The concentration of all constituents in the medium immediately after replenishment are determined by assuming full mixing. In the case of a routine medium replenishment, which simulates the normal experimental procedure for maintaining an adequate level of glucose, a specified volume of medium is exchanged with fresh, with normal glucose level and the specified oxygen level. Note that in an event where the oxygen level is specified, this is both the level in the added medium and the continuing level at the air-medium interface. At the start of every time step the current time is checked against the protocol event times.

After a medium-change event (i.e. a drug dose or medium replenishment) the next time step is subdivided into 12 sub-time steps, in order to improve solver accuracy in a situation where concentrations are changing rapidly.

Radiation

The model is formulated for low linear energy transfer (gamma) radiation providing uniform dose rates throughout the spheroid. If there is a radiation dose in a time step, for each cell that is not already tagged for radiation death the probability of a fatal dose is computed as follows. According to the LQ model for radiation killing, the survival fraction f_S from a radiation dose of D_r Gy^{-1} can be calculated from:

$$-\log_e(f_S) = \alpha_H OER_\alpha D_r + \beta_H (OER_\beta D_r)^2 \quad \text{(S12)}$$

where the oxygen enhancement ratios (*OER*) are given as functions of C_{O2}, the oxygen concentration in the cell, by:

$$\begin{aligned} OER_\alpha(C_{O2}) &= \frac{OER_{\alpha\max} C_{O2} + K_{ms}}{C_{O2} + K_{ms}} \\ OER_\beta(C_{O2}) &= \frac{OER_{\beta\max} C_{O2} + K_{ms}}{C_{O2} + K_{ms}} \end{aligned} \quad \text{(S13)}$$

Input values are: the maximal OER parameters ($OER_{\alpha max}$, $OER_{\beta max}$) determined from radiation survival curves for cells exposed to radiation under anoxic and saturating O_2 conditions, the oxygen concentration at which radiosensitivity is half maximal (K_{ms}), and the LQ parameters determined under anoxia, α_H and β_H. The probability of killing is 1-f_S, therefore for each cell a uniform (0, 1) random variate R is generated and the cell is tagged to die (i.e.is non-clonogenic) from radiation if $R < 1 - f_S$. Tagged cells continue to metabolise and grow. In the default case tagged cells die when they attempt the first cell division. However, tagged cells may proceed through cell division with a probability of death (cytolysis), P_D and also have a cellular growth delay imposed at a user defined rate of *GD* hr/Gy for N_{GD} divisions.

Drug kinetics

While a drug and its metabolites are present in the medium, they are added to the list of constituents for which the reaction-diffusion system of equations given by (S1) – (S8) is solved, with net molar rate functions M_i' as shown in (S15), (S16) and (S17) below. When the concentrations of all the drug compounds fall below a specified threshold they are dropped from the computation.

The model is set up to handle dosing by up to two different drugs, sequentially or concurrently. Each drug is defined by a set of parameters, loaded from a drug data file, including cellular exchange rate constants, K_{in}, K_{out}. Hypoxia-activated drugs typically undergo a sequence of reactions in a cell, and the model allows for two metabolites:

Parent drug ➔ Metabolite 1 ➔ Metabolite 2 ➔ untracked products

The equations that control these three reactions are determined by a set of parameters K_{met0}, F_2, K_{O2}, n, V_{max} and K_m that are specified separately for the parent and up to two metabolites. The rate constant for the maximum rate of metabolism, which for hypoxia-activated prodrugs such as SN30000 occurs when oxygen concentration C_{O2} is zero, is given by

$$K_{met0} + \frac{V_{\max}}{K_m + C},$$

where C is the intracellular drug concentration. The actual reaction rate is the product of this rate constant, C, and a sigmoid function of oxygen concentration C_{O2}, with parameters F_2, K_{O2} and n:

$$R_i' = C\left(1 - F_2 + \frac{F_2 K_{O2}^n}{(K_{O2}^n + C_{O2}^n)}\right)\left(K_{met0} + \frac{V_{\max}}{(K_m + C)}\right) \quad \text{(S14)}$$

With appropriate selection of the parameters this is the rate of transformation of parent drug (i=0) to metabolite 1, or of metabolite 1 to metabolite 2, or removal of metabolite 2. The net molar rate functions inserted into (Eqn 7) become:

$$M_0' = R_0' \quad \text{(S15)}$$

$$M_1' = R_1' - R_0' \quad \text{(S16)}$$

$$M_2' = R_2' - R_1' \quad \text{(S17)}$$

Drug action

The cytotoxic effect can be created by any subset of the three simulated constituents, e.g. in the case of SN30000 only the parent is cytotoxic as a function of its rate of bioreductive metabolism, R_0' , to form a short-lived DNA-damaging free radical [6] while PR104A [7] has no killing effect but both released nitrogen mustard metabolites are cytotoxic. There are five possible drug kill models, reflecting different mechanisms of cytotoxicity, each determined by a killing rate parameter K_d that is estimated from the results of drug killing experiments. For the five models the probability of killing in a small time interval Δt, when the drug concentration is C, is as follows:

model 1: $P_{kill} = K_d R_i' \Delta t$ (S18)

model 2: $P_{kill} = K_d C R_i' \Delta t$ (S19)

model 3: $P_{kill} = K_d (R_i')^2 \Delta t$ (S20)

model 4: $P_{kill} = K_d C \Delta t$ (S21)

model 5: $P_{kill} = K_d C^2 \Delta t$ (S22)

When more than one cytotoxic constituent is present, the resulting probability of kill in the small time interval is determined by evaluating the survival probability as the product of the separate survival probabilities. For two cytotoxic constituents with individual kill probabilities of P_1 and P_2, the combined kill probability is calculated as:

$$P_{kill} = 1 - (1 - P_1)(1 - P_2) \qquad \text{(S23)}$$

Note that "killing" refers to tagging a cell to die at the time of mitosis – tagged cells continue to metabolise and grow until it is time to divide.

Note, for SN30000, we assume that Metabolite 1 is a cytotoxic, short-lived free radical [6] that does not diffuse from the cell, while its more stable downstream 1-oxide and nor-oxide metabolites are non-cytotoxic as demonstrated previously [8–10]. The PD model for SN30000 (Eqn 19), which has previously been validated in single cell suspensions [8,11], describes the probability of clonogenic cell killing (P_{kill}) in a small time interval Δt, as the product of prodrug concentration C and its rate of bioreductive metabolism R' where K_d is the cell killing rate constant. This formulation (and that of Eqn 18 and 20) assumes the rate of generation of the transient intracellular cytotoxic radical is proportional to R', avoiding the need to calculate explicitly the very low intracellular radical concentrations. This PD model (Eqn 19) reflects the hypothesis that SN30000 and other benzotriazine dioxide HAPs can potentiate the initial DNA damage done by the short lived radical [6,12,13] (Figure K).

Program implementation

The model can be run in two ways. For "hands-on" simulations a graphical user interface (GUI) enables the user to give values to parameters of various kinds, to set the initial cell count and the number of days of simulation, to select up to 16 real-time plots from a wide range of options. Alternatively the program can be run from the command-line using a pre-generated input file. The latter mode is also available for executing the program remotely on a high-performance cluster – in this mode it is possible to perform a parameter "sweep", in which runs are submitted with specified combinations of parameter values to be executed in parallel on multiple nodes, and results are retrieved for processing. In all cases the values of all significant variables are written to an output file at 1 hr intervals. Results can also be saved optionally at user-specified intervals and time ranges. The GUI has been developed using Qt Creator (i.e. it is coded in

C++), while the engine, built as a DLL, is coded in Fortran 95. Shared memory parallelisation with OpenMP is employed to take advantage of multiprocessor machines.

When the program is executed interactively the run parameters selected in the GUI are saved to an input file, the name of which is passed to the simulation engine. The execution thread in the GUI program then enters a loop in which a DLL procedure is called to advance the simulation through a time step, and various procedures are called to retrieve results for display in other threads. Asynchronous status and error messages are sent to the GUI for display via a socket using IP. The graphical displays available to the user are of six kinds: (a) time-series plots of all state variables of interest, (b) profile plots of intracellular (IC) and extracellular (EC) constituent concentrations (value of a selected variable on a line through the centre of the spheroid), (c) 2D plot of EC concentrations, within the fine grid, and the cells (value of a selected variable on a specified plane), (d) zoomable, rotateable 3D rendition of the spheroid cells (using VTK/OpenGL), (e) Flow cytometry plots of IC concentration, (f) probability histogram plots of IC concentration. Figure D illustrates the range of displays. These plots are all updated as the program executes. Figure E shows the plot screen, which can be scrolled to view up to 16 time-series or profile plots, selected from a menu of 35 time-series options and 23 profile options. Optionally, the user can choose to create videos of the flow cytometry plot, the histogram plot, and the 3D rendition. A comprehensive set of result values is written to an output file after each time step, for post-processing.

Alternatively, the simulation can be run without the GUI. In this case the input file must be provided, and in the main program the executing loop simply calls the subroutines that advance the simulation through a time step and write out the results. This is the mode of execution used in production runs, typically on a 'supercomputer' cluster. To facilitate the queueing up of multiple simulation runs in which parameters are varied (e.g. for calibration purposes) a

spreadsheet has been developed, in which the user can specify which input parameters are to be varied, over what range and with what step, then submit the set of runs to the cluster. The output files for each run are returned automatically on run completion into separate directories on the user's PC.

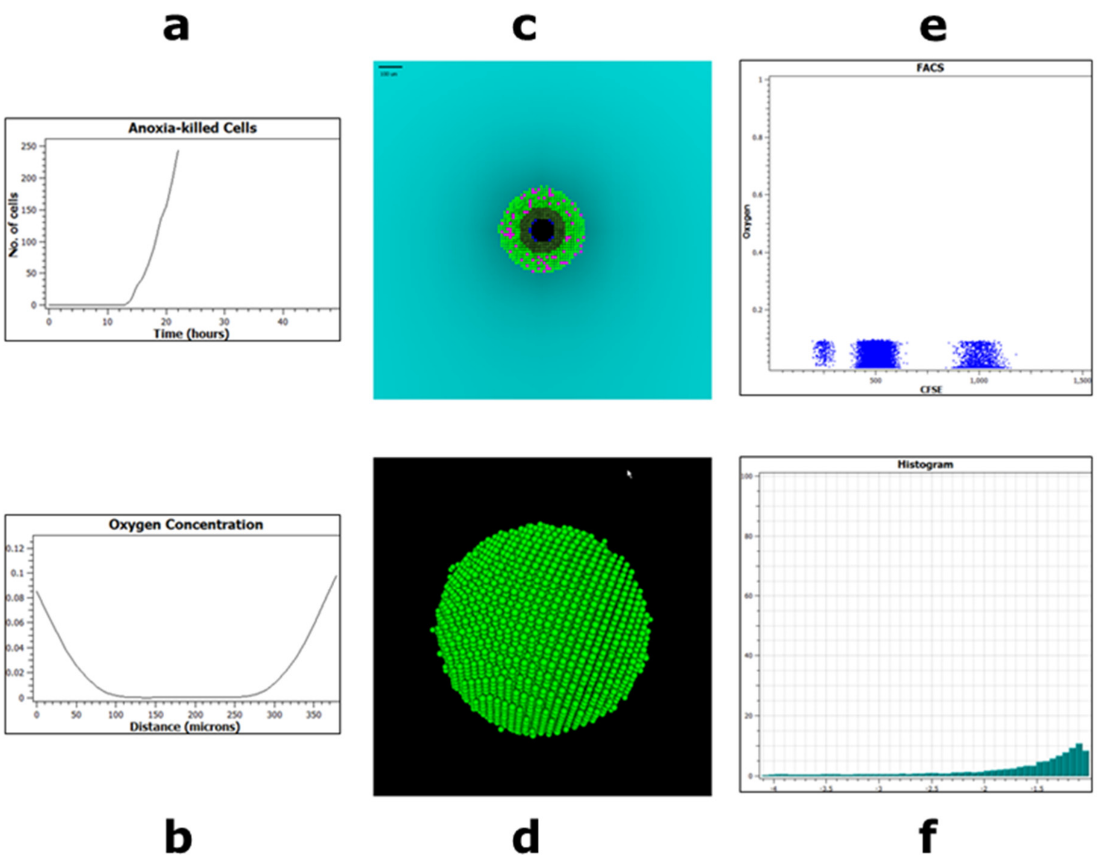

Figure D. The range of real-time graphical displays of spheroid state available in the GUI. (**a**) time-series plot of number of cells killed by hypoxia, (**b**) profile plot of EC oxygen concentration within the spheroid, (**c**) 2D plot of oxygen concentration in the fine grid, light green cells are oxic, dark green cells are hypoxic (< 1μM O_2), blue cells are tagged to die, pink cells are in mitosis, black region is necrotic, (**d**) 3D rendition of spheroid, (**e**) Flow cytometry plot of oxygen vs. CFSE (a dye that indicates cell generation), (**f**) log-scale histogram of IC oxygen level.

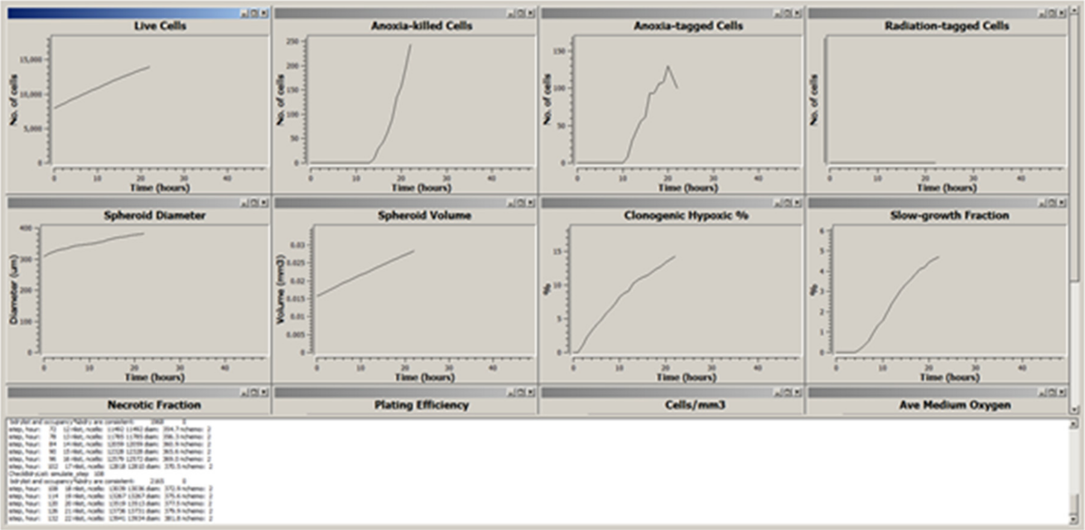

Figure E. The main GUI results screen, showing 8 of the 32 available plots.

Monolayer agent-based model

The MABM, to simulate experiments in monolayer cultures, is based on three simplifying (and related) assumptions:

1. Concentrations of all medium constituents depend only on depth (on the *z* coordinate), i.e. there is no dependence on the lateral position (*x*, *y*). This implies a 1D formulation for concentrations.

2. Since all cells are at the same depth, they are all exposed to the same concentrations of nutrients and drugs.

3. The current formulation of cellular metabolism for these cancer cells (in this model and in the spheroid ABM) does not include dependence on cell cycle stage – rates of consumption of oxygen and glucose depend only on the local concentrations of these constituents. Similarly, intracellular drug reaction rates depend only on intracellular oxygen concentration, therefore at any instant all cells have the same intracellular levels of drug and metabolites.

The combination of these assumptions makes it feasible to solve the intracellular reactions for a single cell, using the same parameter values as the spheroid ABM, then use the concentrations for all cells. This leads to a great increase in speed of execution of the model. From the start of the simulation cells are at different points in the cell cycle (through randomisation of initial cell size). As a consequence although all cells have the same rates of metabolism, and the same rate of volume growth, cell division and death is not synchronized.

Solving for concentrations

The solvers for oxygen, glucose and for drugs follow a similar pattern. The medium depth is subdivided into a number of layers (e.g. $NZD = 20$), and the solvers determine the concentrations in each layer, and the intracellular concentrations, in each time step. For oxygen and glucose, the 1D PDE in the medium is converted into a set of ODEs (one for each layer), which is supplemented by the ODE for the intracellular concentration. Mass flux at the lower boundary of the medium grid is given by the rate of uptake of oxygen or glucose by an individual cell multiplied by the number of live cells, and inter-layer mass flux is proportional, through the diffusion coefficient, to the concentration difference. The set of ODEs is solved using the variable time-step Runga-Kutta solver RKC.

In the case of a drug, in general the code allows for three constituents – the parent drug, and two metabolites. The method of solution is the same as for oxygen or glucose, but depending on the drug there can be up to three concentrations in each layer and in the representative cell. Note that drug metabolism reactions occur only within a cell, while instability (first order decay) can occur anywhere. Depending on the relative concentrations in the cell and in the bottom medium layer, the flux of a drug and its metabolites can be either uptake or release.

The upper boundary is a wall (no flux) for glucose and drugs, while in the case of oxygen the gas-phase concentration has the user-specified fixed value.

In each time step, the updating of the constituent concentrations is carried out sequentially, except that in the case of a drug the intracellular reactions for the drug and metabolites are handled together. By setting diffusion coefficients to very high values, the MABM can be used to simulate stirred single-cell suspensions.

Parameter Estimation

A simple "grid-search" method was developed, using monolayer model simulations of monolayer experiments to estimate selected model parameters. The procedure uses iterative search optimisation to find the parameter values that provide the best fit to a set of experiments. The user specifies the parameters to be varied, the range of variation and number of values of each, and the experimental observables that will be used to determine goodness of fit. For a given set of parameter values model runs are carried out to simulate each experiment, and the objective function is evaluated as the weighted sum of squares of errors, where the error is the difference between the simulated value and the experimentally measured value. All combinations of the parameter values are simulated and the parameter combination that minimises the objective function is identified. This process is carried out through a specified number of iterations, where at each iteration the range of variation of each parameter about its current best value is reduced. Because the number of parameter combinations can become very large, the fitting is usually carried out for only a small subset of the model parameters, the rest being held fixed at their measured or assumed values during the experiment. The user interface for the fitter is an Excel spreadsheet.

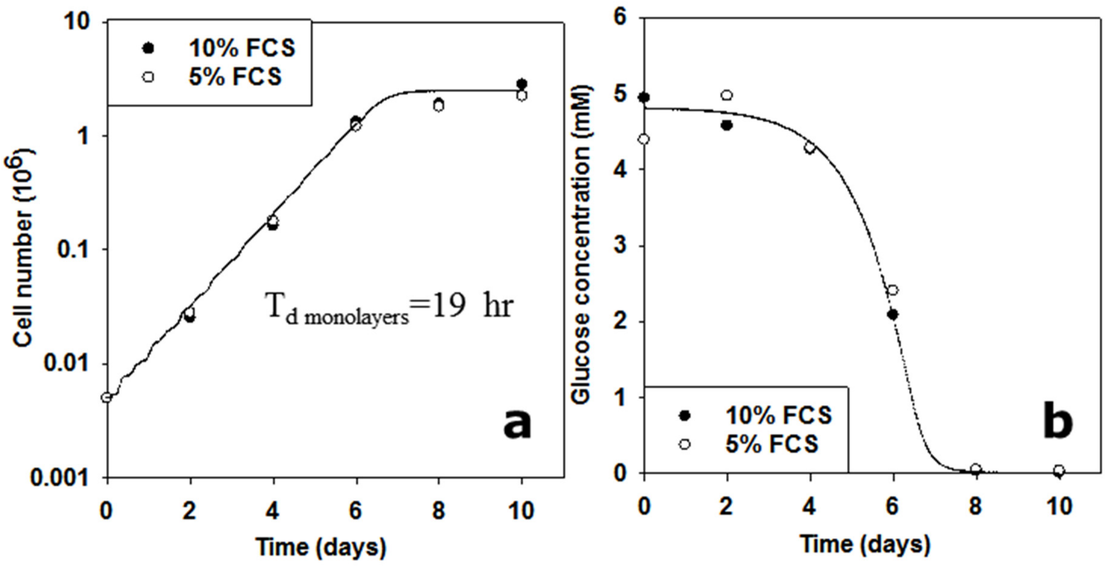

Figure F. HCT116 monolayer growth (a) and glucose consumption (b). The MABM was used to estimate the doubling time, T_d, based on observation of HCT116 monolayer growth. HCT116 monolayers (5×10^3 cells/well) in 6-well plates with 4 mL of αMEM supplemented with 10% or 5% FCS were cultured in 20% O_2/5% CO_2 humidified incubator without medium replenishment. Cell number and glucose concentrations in specific wells were measured. Lines are model fits to the cell count and glucose concentration data. $T_{d\ monolayers}$ was the fitted parameter with glucose metabolism parameters fixed at the estimated values in Table 1.

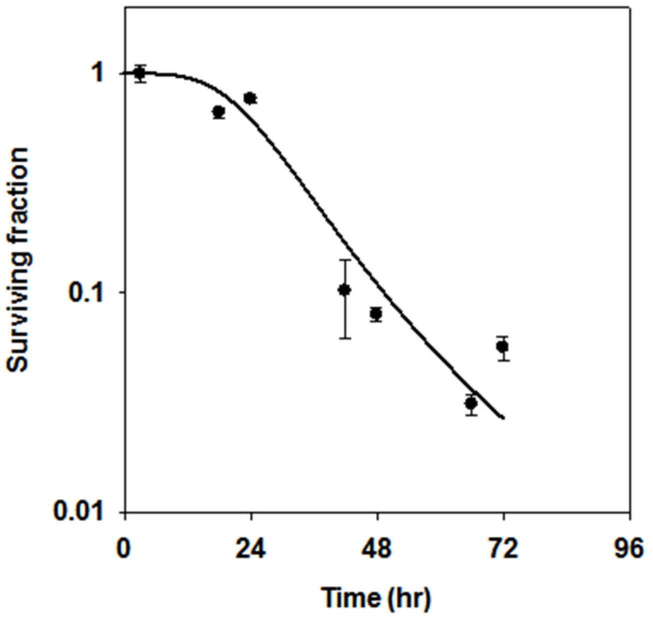

Figure G. Survival of HCT116 cells under anoxia. HCT116 monolayers (2×10^4 cells) in 6-well plates with 4 mL of αMEM+5% FCS were exposed to anoxia at 37°C (anoxic chamber) for the indicated times before dissociation, counting and plating for clonogenic survival assay. Points are mean ± SEM for 3 replicates.

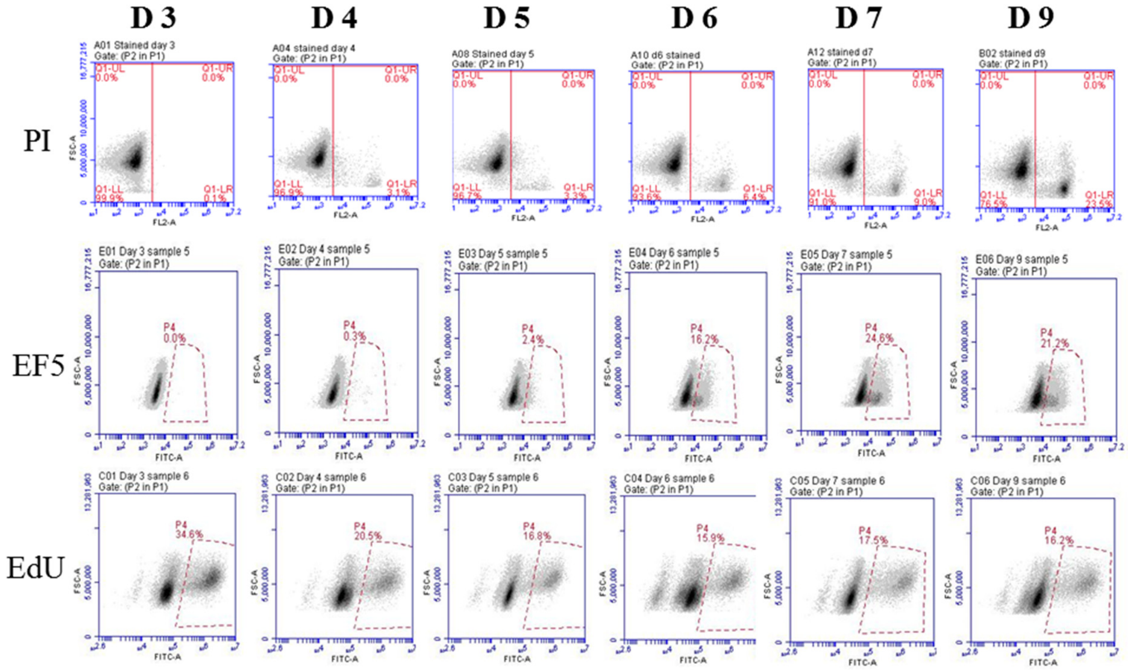

Figure H. Quantitation of cellular characteristics of HCT116 spheroids by flow cytometry. Representative scatter plots of cell viability (% PI negative), hypoxic fraction (% EF5-positive cells) and S-phase fraction (% EdU-positive cells) for day 3 - day 9 spheroids. Summary data are shown in Figure 5.

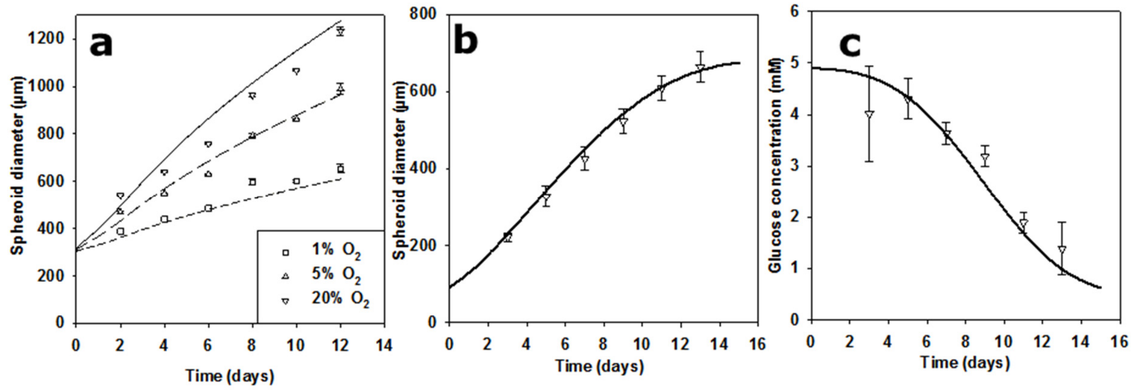

Figure I. Oxygen dependence and un-fed spheroid growth and comparison with the SABM. (**a**) HCT116 spheroids (seeded with 2×10^3 cells/well) were cultured under 20%, 5% or 1% O_2 and the diameters of spheroids were monitored (points) during medium change every 2nd day and simulated (lines) as a function of time. Simulations are based on the model parameters in Table S1. Experimental values are means ± SD for 4 replicates. (**b, c)** HCT116 spheroids (seeded with 10^3 cells/well) were cultured in glucose-free DMEM with 10% FCS supplemented with an initial concentration of 5 mM D-glucose without replacement of the medium. Spheroid diameter (points in **b**) was measured on the indicated days, as was the concentration of D-glucose in medium (points in **c**). Values are means ± SD for 4 replicates. The SABM simulations, based on model parameters in Table S1 show good agreement with experimentally determined spheroid growth (lines in **b**) and consumption of D-glucose in medium (lines in **c**).

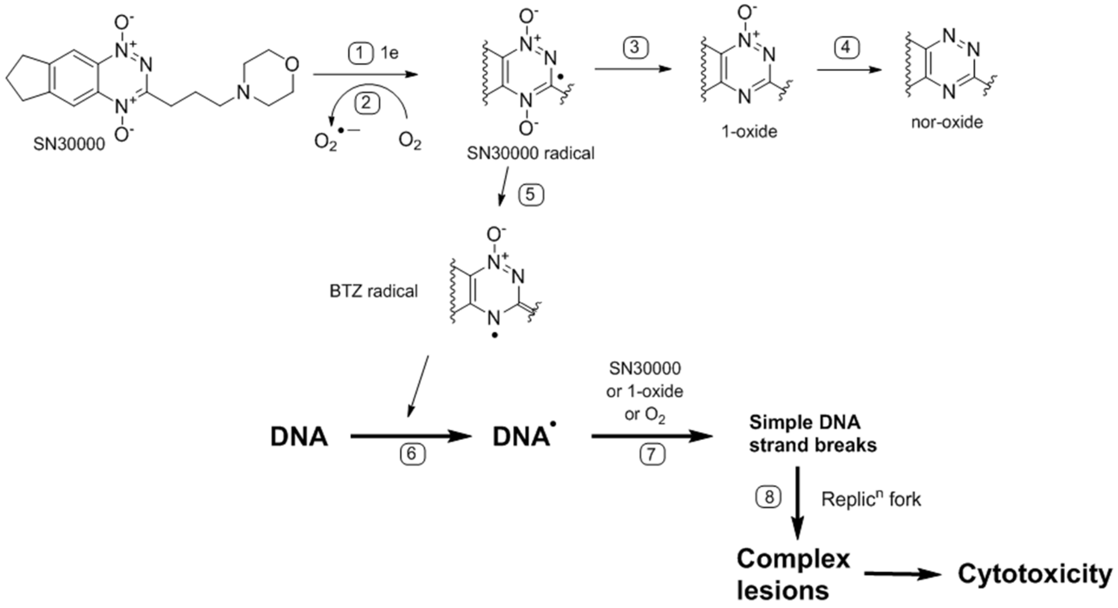

Figure J. SN30000 metabolism by 1-electron reductases and proposed mechanism of cytotoxicity. SN30000 is metabolised by 1-electron reductases (1) to an initial radical which is re-oxidised to SN30000 in the presence of O_2 (2) providing hypoxic selectivity. The initial radical may undergo further reduction to the 2 electron of 4 electron reduction products (1-oxide and nor oxide, steps 3 & 4) or formation of an oxidising benzotriazinyl radical capable of causing initial DNA damage. These radical anions are short lived and retained within the cell of origin. It is proposed that SN30000, its 1-oxide or oxygen can oxidise the initial DNA radical (7) resulting in strand breaks that then become complex DNA lesions. For more details see [6,12,13]

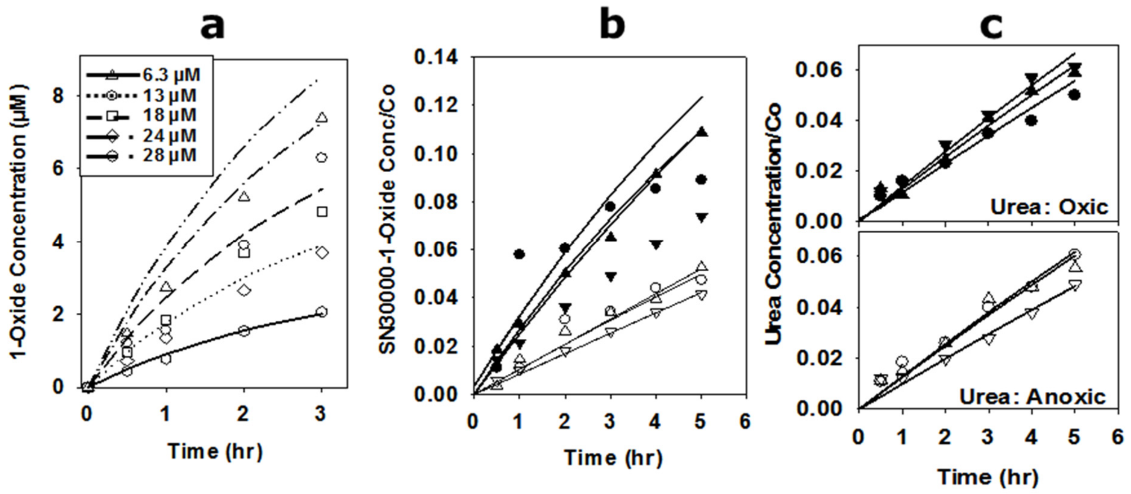

Figure K. Development of a spatially resolved PK/PD model for SN30000. Supplementary to the data in Figure **6**, bioreductive metabolism of SN30000 under anoxia was confirmed by the appearance of SN30000-1-oxide in medium (**a**) in anoxic stirred single cell suspensions, and in the donor (**b**, filled symbols) and receiver (**b**, open symbols) compartments in MCL experiment for determining SN30000 diffusion with predictions assuming 75% conversion to SN30000-1-oxide. Each MCL in Figure 6 was of similar thickness as estimated from diffusion of ^{14}C-urea (**c**).

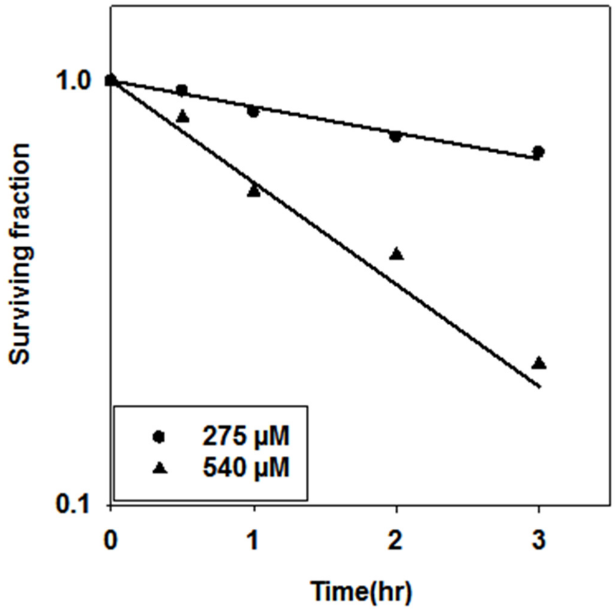

Figure L. Cell killing by SN30000 in stirred cell suspensions under 20% O_2 at 2 initial SN30000 concentrations. Lines are model fits using the MABM assuming the medium was fully stirred, that oxygen effects the rate of metabolism according to Eqn (S14) and the same PD model as used under anoxia in Figure 6 (Model 2, Eqn (S19)). The fitted parameter was the Hill coefficient for oxygen dependence of SN30000 metabolism in Eqn (S19).

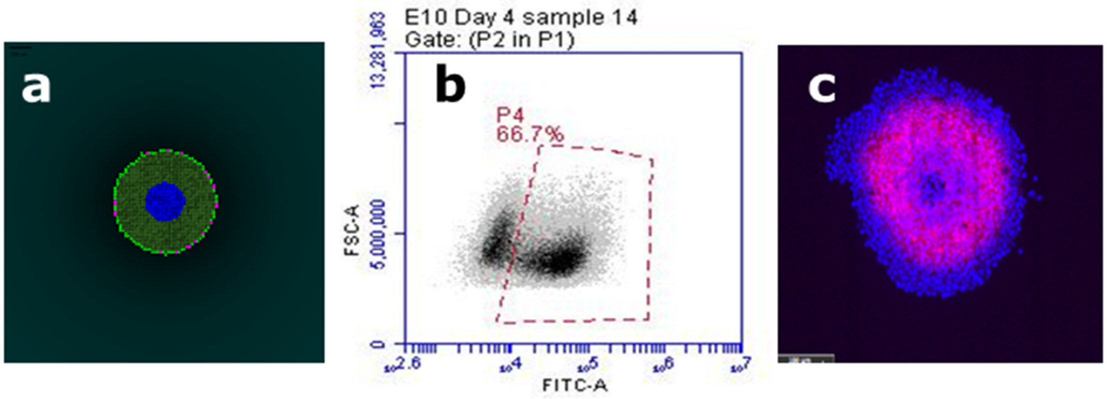

Figure M. Hypoxic fraction of spheroids under 5% O_2. The SABM predicted higher hypoxic fraction of spheroids (with diameter ca. 450 μm) pre-incubated under 5% O_2 for 3 hr (**a**) than that under 20% O_2 (Figure 3C). To confirm this experimentally, 4 day HCT116 spheroids pre-incubated under 5% O_2 for 3 hr were then exposed to hypoxia probe EF5 for 2 hr, followed by dissociating spheroids to single cell suspensions for flow cytometry (**b**) or by fixing and sectioning spheroids for immunostaining (**c**).

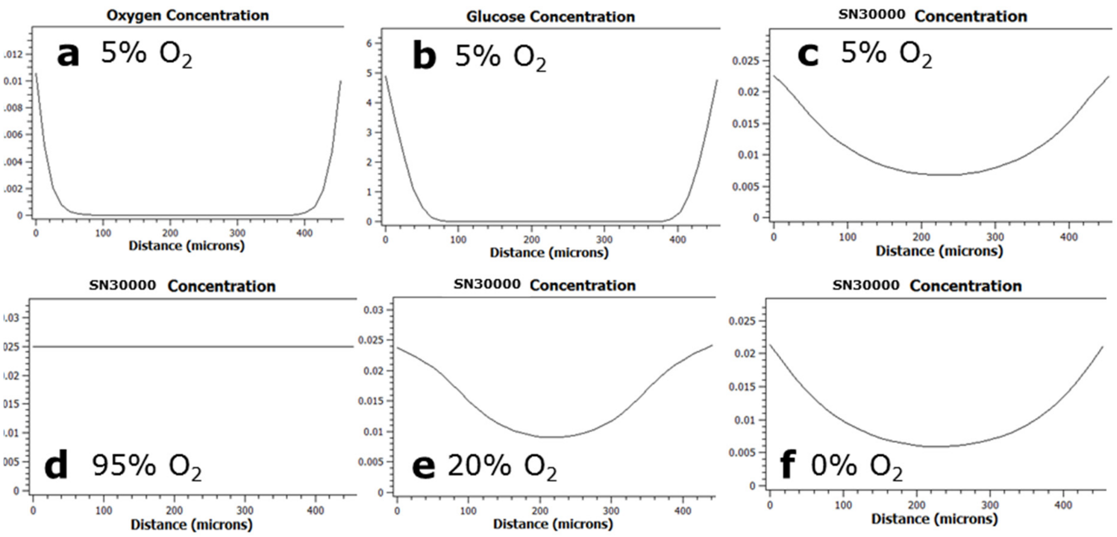

Figure N. Upper Row: O_2 (**a**), glucose (**b**) and SN30000 (**c**) concentration profiles under treatment conditions when spheroids (ca. 460 μm) were exposed to SN30000 under 5% O_2. Lower Row (**d-f**): Predicted concentration profiles for SN30000 exposure under the indicated oxygen conditions are shown for comparison.

Supporting Information References

1. Ruuth SJ (1995) Implicit explicit methods for reaction-diffusion problems in pattern formation. J Math Biol 34: 148-176. Doi 10.1007/Bf00178771.

2. Saad Y, Schultz MH (1986) Gmres - a Generalized Minimal Residual Algorithm for Solving Nonsymmetric Linear-Systems. Siam J Sci Stat Comp 7: 856-869. Doi 10.1137/0907058.

3. Saad Y (2006) ITSOL - a library of iterative solvers for general sparse linear systems of equations. http://www-users.cs.umn.edu/~saad/software/ITSOL/.

4. Sommeijer BP, Shampine LF, Verwer JG (1997) RKC: an explicit solver for parabolic pdes. technical report mas-r9715, cwi, amsterdam.

5. Sommeijer BP, Shampine LF, Verwer JG (1998) RKC: An explicit solver for parabolic PDEs. J Comput Appl Math 88: 315-326. Doi 10.1016/S0377-0427(97)00219-7.

6. Anderson RF, Yadav P, Patel D, Reynisson J, Tipparaju SR, Guise CP, Patterson AV, Denny WA, Maroz A, Shinde SS, Hay MP (2014) Characterisation of radicals formed by the triazine 1,4-dioxide hypoxia-activated prodrug SN30000. Ogranic and Biomolecular Chemistry 12: 3386-3392.

7. Foehrenbacher A, Secomb TW, Hicks KO, Wilson WR (2012) The contribution of metabolite diffusion to the anti-tumour activity of the hypoxia-activated prodrug PR-104A.

8. Hicks KO, Siim BG, Jaiswal JK, Pruijn FB, Fraser AM, Patel R, Hogg A, Liyanage HDS, Dorie MJ, Brown JM, Denny WA, Hay MP, Wilson WR (2010) Pharmacokinetic/pharmacodynamic modeling identifies SN30000 and SN29751 as tirapazamine analogues with improved tissue penetration and hypoxic cell killing in tumors. Clin Cancer Res 16: 4946-4957.

9. Wang J, Guise CP, Dachs GU, Phung Y, Hsu AH, Lambie NK, Patterson AV, Wilson WR (2014) Identification of one-electron reductases that activate both the hypoxia prodrug SN30000 and diagnostic probe EF5. Biochem Pharmacol 91: 436-446. S0006-2952(14)00458-4 [pii];10.1016/j.bcp.2014.08.003 [doi].

10. Gu Y, Chang TTA, Wang J, Jaiswal JK, Edwards D, Downes NJ, Liyanage HDS, Lynch C, Pruijn FB, Hickey AJR, Hay MP, Wilson WR, Hicks KO (2017) Reductive Metabolism Influences the Toxicity and Pharmacokinetics of the Hypoxia-Targeted Benzotriazine Di-Oxide Anticancer Agent SN30000 in Mice. Frontiers in Pharmacology 8: 531. 10.3389/fphar.2017.00531.

11. Hicks KO, Pruijn FB, Secomb TW, Hay MP, Hsu R, Brown JM, Denny WA, Dewhirst MW, Wilson WR (2006) Use of three-dimensional tissue cultures to model extravascular transport and predict in vivo activity of hypoxia-targeted anticancer drugs. J Natl Cancer Inst 98: 1118-1128.

12. Siim BG, Pruijn FB, Sturman JR, Hogg A, Hay MP, Brown JM, Wilson WR (2004) Selective potentiation of the hypoxic cytotoxicity of tirapazamine by its 1-N-oxide metabolite SR 4317 . Cancer Res 64: 736-742.

13. Hunter FW, Wang J, Patel R, Hsu HL, Hickey AJ, Hay MP, Wilson WR (2012) Homologous recombination repair-dependent cytotoxicity of the benzotriazine di-N-oxide CEN-209: Comparison with other hypoxia-activated prodrugs. Biochem Pharmacol 83: 574-585. S0006-2952(11)00899-9 [pii];10.1016/j.bcp.2011.12.005 [doi].
